# High diversity of the Ganzhou Oviraptorid Fauna increased by a new “cassowary-like” crested species

**DOI:** 10.1038/s41598-017-05016-6

**Published:** 2017-07-27

**Authors:** Junchang Lü, Guoqing Li, Martin Kundrát, Yuong-Nam Lee, Zhenyuan Sun, Yoshitsugu Kobayashi, Caizhi Shen, Fangfang Teng, Hanfeng Liu

**Affiliations:** 1 Institute of Geology, Chinese Academy of Geological Sciences, Beijing, 100037 China; 2 Key Lab of Stratigraphy and Paleontology, Ministry of Land and Resources of China, Beijing, 100037 China; 3 Jiangxi College of Applied Technology, Ganzhou, 341000 Jiangxi Province China; 4 Center for Interdisciplinary Biosciences, Faculty of Science, University of Pavol Jozef Safarik, Kosice, 04154 Slovak Republic; 5 School of Earth and Environmental Sciences, Seoul National University, Seoul, 08826 South Korea; 6 Jinzhou Paleontological Museum, Jinzhou, 121000 Liaoning Province China; 7 Hokkaido University Museum, Hokkaido University, Sapporo, Hokkaido, 060-0810 Japan; 8 Xinghai Paleontological Museum of Dalian, Dalian, 116000 Liaoning Province China

## Abstract

A new oviraptorid dinosaur from the Late Cretaceous of Ganzhou, bringing oviraptrotid diversity of this region to seven taxa, is described. It is characterized by a distinct cassowary-like crest on the skull, no pleurocoels on the centra from the second through fourth cervical vertebrae, a neck twice as long as the dorsal vertebral column and slightly longer than the forelimb (including the manus). Phylogenetic analysis recovers the new oviraptorid taxon, *Corythoraptor jacobsi*, as closely related to *Huanansaurus* from Ganzhou. Osteochronology suggests that the type specimen of *Corythoraptor* had not reached stationary growth stage but died while decreasing growth rates. The histology implies that it would correspond to an immature individual approximately eight years old. We hypothesize, based on the inner structure compared to that in modern cassowaries, that the prominent casque of *Corythoraptor* was a multifunction-structure utilized in display, communication and probably expression of the fitness during mating seasons.

## Introduction

Oviraptorosaurs, a well-defined group of coelurosaurian dinosaurs, are characterized by short, deep skulls with toothless jaws (teeth are present in primitive forms such as *Incisivosaurus* and *Caudipteryx*), pneumatized caudal vertebrae, anteriorly concave pubic shafts, and posteriorly curved ischia^1–3^. In recent years, diverse oviraptorid-like eggs (and clutches) as well as oviraptorid skeletons that have been unearthed from the Upper Cretaceous deposits of Ganzhou, Jiangxi Province, southern China, have made the Ganzhou area one of the most productive oviraptorosaurian regions of the world. At present, six oviraptorosaurian dinosaurs have been named from Ganzhou, including *Banji long* ^4^, *Jiangxisaurus ganzhouensis* ^5^, *Nankangi*a *jiangxiensis* ^6^, *Ganzhousaurus nankangensis* ^7^, *Huanansaurus ganzhouensis* ^8^, and *Tongtianlong limosus* ^9^. All taxa are from the Upper Cretaceous Nanxiong Formation, comprising a distinct “Ganzhou Dinosaurian Fauna”^8^. Here we describe a new oviraptorid dinosaur *Corythoraptor jacobsi* gen. et sp. nov. from the Nanxiong Formation beds exposed near the Ganzhou Railway Station, Ganzhou City, Jiangxi Province. *Corythoraptor jacobsi* gen. et sp. nov. bears a distinct cassowary-like crest (helmet) and has a long neck, which exhibits convergent morphology to the modern flightless cassowary bird from Queensland in Australia. The discovery of *Corythoraptor jacobsi* provides unprecedented evidence that oviraptorid dinosaurs were morphologically and taxonomically far more diverse in the Ganzhou area than in any other known region of the world.

## Results

### Systematic palaeontology

Oviraptorosauria Barsbold, 1976.

Oviraptoridae Barsbold, 1976.

*Corythoraptor jacobsi* gen. et sp. nov.

### Etymology

The generic name *Corythoraptor* refers to a raptor bearing a “cassowary-like crest” on its head, and the specific name is in honor of Professor Louis L. Jacobs, who has contributed to dinosaur research and has given excellent mentoring to three authors (JLü, YL and YK) when they were Ph.D. students at Southern Methodist University, Dallas, Texas, USA.

### Holotype

**Figure 1:**
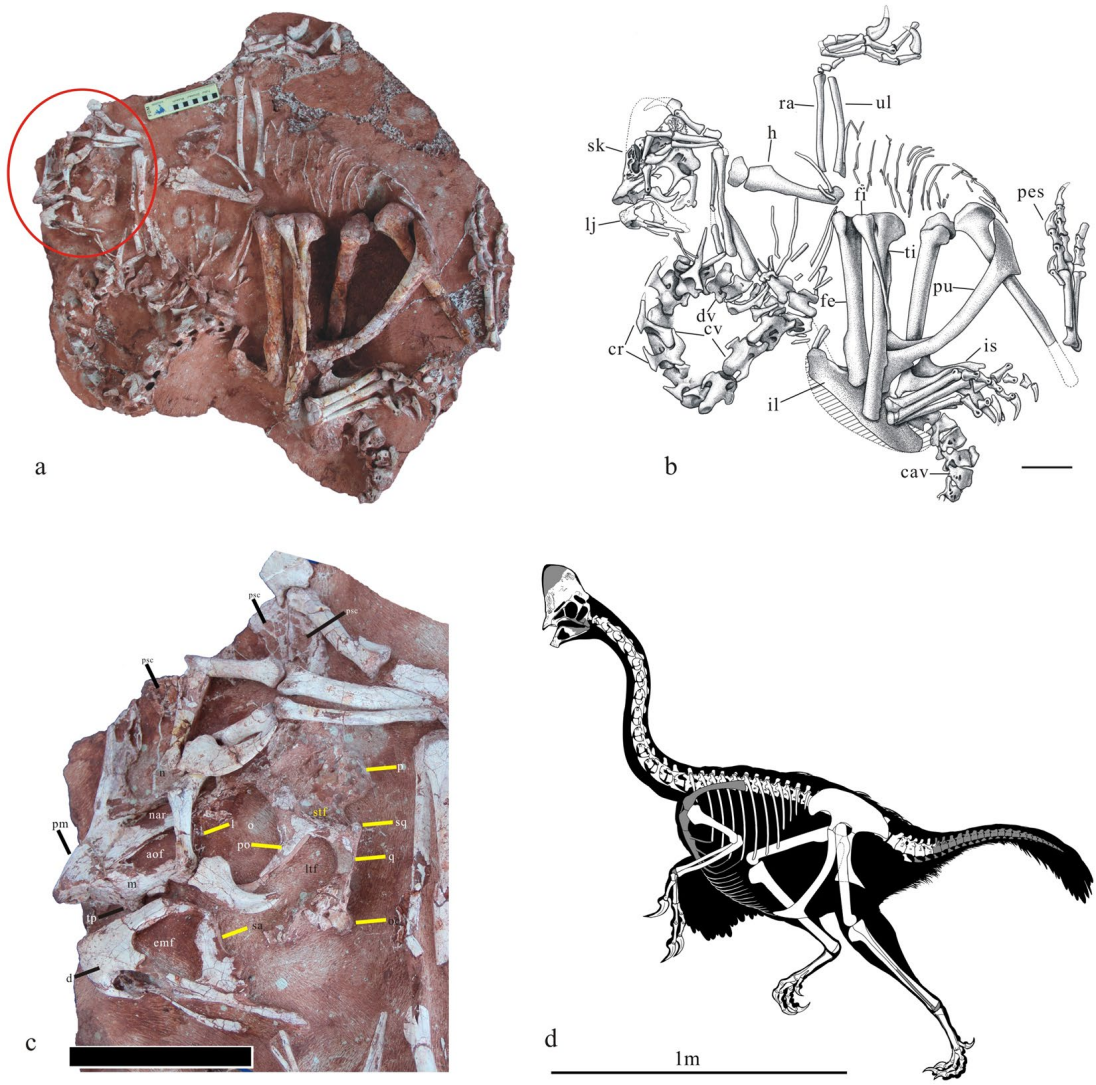
The holotype of *Corythoraptor jacobsi* gen. et sp. nov. (JPM-2015-001). (**a**) Photograph. (**b**) Outline drawings. (**c**) Close up of the skull and lower jaw, showing the pneumatic cassowary-like crest (Only skull and lower jaw elements are labeled). (**d**) Skeletal reconstruction (missing parts are in grey). Abbreviations: aof, antorbital fenestra; cav. caudal vertebrae; cr. cervical ribs; cv. cervical vertebrae; dv. dorsal vertebrae; fe, femur; fi. fibula; h, humerus; il, ilium; is, ischium; l, lacrimal; lj, lower jaw; ltf, lower temporal fenestra; m, maxilla; n, nasal; nar, narial opening; o, orbit; oc, occipital condyle; p, parietal; pm, premaxilla; po, postorbital; ps, pes; psc, pneumatic skull crest; pu. pubis; q, quadrate; ra, radius; sk, skull; sq, squamosal; stf, super temporal fenestra; ti, tibia; ul, ulna. Scale bar = 8 cm in (**c**) and 100 cm in (**d**).

**Figure 2:**
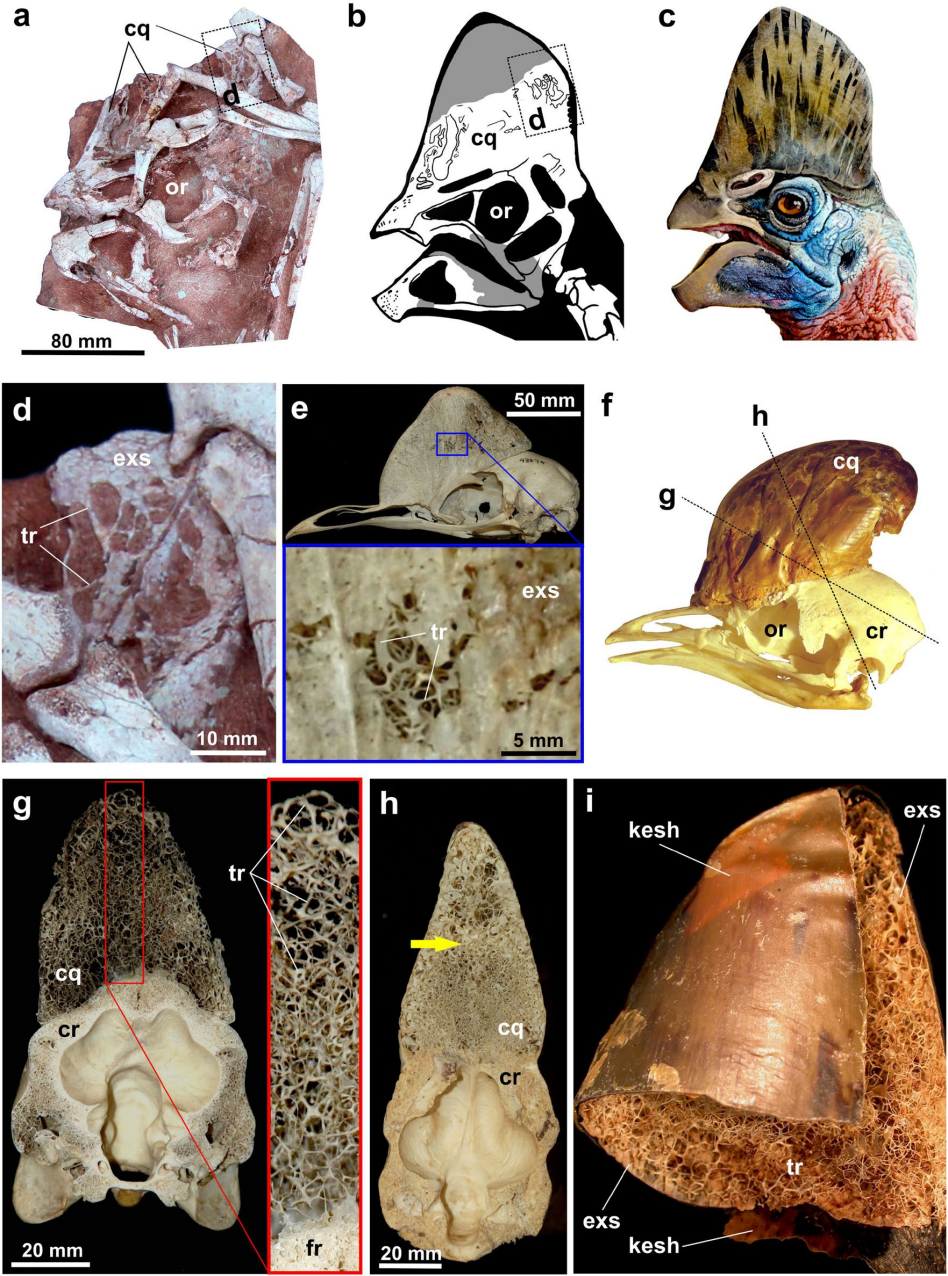
The cranial casque of *Corythoraptor jacobsi* and recent cassowaries. (**a**–**c**) the crested skull of *Corythoraptor* and head appearance restorations. (**d**) a close-up (see dotted rectangle in **a** and **b**) of eroded bony shell in the posterolateral casque of *Corythoraptor*. (**e**) the crested skull of the recent cassowary (*Casuarius uniappendiculatus*; Museum für Naturkunde in Berlin, Germany: MfN-ZMB 93274). (**f**) a keratinous helmet over the skull of the recent cassowary (unnumbered specimen of *Casuarius casuarius* from the osteological collections of ZOO Protivín, Czech Republic). (**g**,**h**) coronal cuts through the cassowary skull – (**g**) *Casuarius casuarius* MfN-ZMB 36820, (**h**) *Casuarius casuarius*: MfN-ZMB 36885 (see dotted lines in **f**); note transition in strut-like trabecular arrangement. (**i**) close-up to contact between keratinous and skeletal components of the casque in recent cassowary, unnumbered specimen of *Casuarius* sp. from the osteological collections of Field Museum of Natural History, Chicago, USA. Abbreviations: cq, casque; cr, cranium; exs, external surface; kesh, keratinous sheath; or, orbit; tr, trabeculae.

Almost complete skeleton with the skull and lower jaw (JPM-2015-001) (Figs 1 and 2) is housed at the Jinzhou Paleontological Museum, Jinzhou, Liaoning Province, China.

### Type locality and horizon

A site in the vicinity of the Ganzhou Railway Station (GPS coordinates are provided on request from the first author), Ganzhou City; Campanian-Maastrichtian; Nanxiong Formation (Upper Cretaceous)^10^.

### Diagnosis

An oviraptorosaurian dinosaur with the following unique combination of characters: ratio of the length of the tomial margin of the premaxilla to the premaxilla height (ventral to the external naris) is 1.0–1.4; inclination of the anteroventral margin of the premaxilla relative to the horizontally positioned ventral margin of the jugal posterodorsal; antorbital fossa bordered anteriorly by the maxilla; narial opening much longer than width; infratemporal fenestra dorsoventrally elongate, narrow anteroposteriorly; the supranarial process of the premaxilla bears two processes: a short posterodorsally extending process, forming the anterodorsal margin of the external nasal opening, and a long process, forming most of the anterodorsal process of the premaxilla; distinct cassowary-like helmet on the skull; long axis of the external narial opening parallel to the dorsal margin of antorbital fenestra; straight anterodorsal margin of dentary in lateral view; a deep fossa, sometimes with associated pneumatopore on lateral surface of dentary; no pleurocoels on the centra from the second through fourth cervical vertebrae; the length of the neck twice as long as the dorsal vertebral column, and slightly longer than the entire forelimb length (including the manus); less pronounced deltopectoral crest of humerus, forming an arc rather than being quadrangular; ratio of the length of the manus to the length of the humerus plus the radius between 0.50 and 0.65; the ungual of digit III less curved than other unguals; lesser trochanter (cranial trochanter) completely fused with the greater trochanter and distal ends of shafts of metatarsal II straight and metatarsal IV laterally deflected.

*Corythoraptor jacobsi* gen. et sp. nov. is assigned to oviraptorid dinosaurs based on the following characters: proximal caudals with pneumatized centra; ischium with its posterior profile concave^3^; premaxilla pneumatized; the subantorbital portion of the maxilla inset medially; the palate extending below the cheek margin; the external naris overlapping most of the antorbital fossa rostrodorsally; the bones of the skull roof pneumatized; the pubic shaft concave cranially, the mandibular symphysis tightly sutured; the shortened preorbital region, and the toothless jaws^2^.

*Corythoraptor jacobsi* differs from all other oviraptorosaurs containing skulls, such as *Incisivosaurus gauthieri* ^11^, *Caudipteryx zoui* ^12^, *Conchoraptor gracilis* ^13^, *Wulatelong gobiensis* ^14^, *Banji long* ^4^, *Khaan mckennai* ^15^, *Citipati osmolskae* ^15, 16^, *Huanansaurus ganzhouensis* ^8^, *Yulong mini* ^17^, *Oviraptor philoceratops* ^16, 18^, *Nemegtomaia barsboldi* ^19, 20^, *Rinchenia mongoliensis* ^21^ ( = *Oviraptor mongoliensis* ^13^) and *Heyuannia huangi* ^22^, in that *Corythoraptor jacobsi* bears a cassowary-like, very thin highly pneumatic cranial crest, the highest point of the crest projecting above the orbit, and an elongated narial opening. Although *Banji long*, *Citipati osmolskae*, *Oviraptor philoceratops*, *Nemegtomaia barsboldi* and *Rinchenia mongoliensis* also bear crests, their morphology and positions at the skull are quite different from that of *Corythoraptor*.

*Corythoraptor jacobsi* differs from *Heyuannia huangi* ^22^ in that *Heyuannia huangi* has no skull crest, the pneumatic foramina on the neural arches and ribs of cervical vertebrae, no infradiapophyseal fossa in the anterior caudal vertebrae, the strongly reduced metacarpal III, and the length ratio 1.25 of tibia to femur, whilst *Corythoraptor jacobsi* does not have.

*Corythoraptor jacobsi* differs from *Shixinggia oblita* ^23^ in that *Shixinggia oblita* has the preacetabular process of the ilium distinctively shorter than the postacetabular process of the ilium, the distal end of the preacetabular process higher than the dorsal margin of the acetabulum, the ischial peduncle almost the same depth with that of the pubic peduncle, and a large opening in the medial surface near the proximal end of the femur.

*Corythoraptor jacobsi* differs from *Jiangxisaurus ganzhouensis* ^5^, which has a weakly downturned mandibular symphysis, pleurocoels in all cervical vertebrae except atlas, the radius-humerus length ratio of about 70%, and slender metacarpal III.

*Corythoraptor jacobsi* differs from *Ganzhousaurus nankangensis* ^7^, which has an acute angle formed by the mandibular symphysis and the dorsal margin of dentary, and a relatively short and nearly straight first pedal ungual.

*Corythoraptor jacobsi* mainly differs from *Huanansaurus ganzhouensis* ^8^ in the skull morphology and the structure of the cervical vertebrae: *Huanansaurus ganzhouensis* has no skull crest, the anterior margin of the premaxilla is nearly vertical to the ventral margin of the skull, the posterior margin of the lower temporal fenestra is oblique, and the dorsal margin of dentary above the external mandibular fenestra is strongly concave ventrally.

*Corythoraptor jacobsi* differs from *Tongtianlong limosus* ^9^ in the skull morphology. The anteroventral corner of the external naris is far above a horizontal line tangent with the posterodorsal corner of the antorbital fenestra, and the skull is dome-like in *Tongtianlong*. Whist the anteroventral corner of the external naris is below a horizontal line tangent with the posterodorsal corner of the antorbital fenestra, and the skull bears a large cassowary-like helmet in *Corythoraptor*.

*Corythoraptor jacobsi* also differs from *Wulatelong gobiensis* ^14^ in that the anteroventral corner of the external naris is far below a horizontal line tangent with the posterodorsal corner of the antorbital fenestra, whilst the anteroventral corner of the external naris is slightly below a horizontal line tangent with the posterodorsal corner of the antorbital fenestra in *Corythoraptor jacobsi*; the ischium/pubis ratio length is much smaller in *Wulatelong gobiensis* (IS/PU = 0.45) than in *Corythoraptor jacobsi* (0.61) and the tibia/femur length ratio is 1.10 in *Wulatelong gobiensis*, which is smaller than that in *Corythoraptor jacobsi* (1.19).

*Corythoraptor jacobsi* differs from *Machairasaurus leptonychus* ^24^, which has elongate and blade-like manual unguals I–III in lateral view, metacarpal I is proportionately short, about 41 per cent the length of metacarpal II, which is shorter than that of *Machairasaurus leptonychus* (about 50% the length of metacarpal II; the combined length of phalanges II-1 and II-2 is about 133% the length of metacarpal II in *Corythoraptor jacobsi*, which is larger than that of *Machairasaurus leptonychus* ^24^, where combined length of phalanges II-1 and II-2 does not exceed 110% the length of metacarpal II.

*Corythoraptor jacobsi* differs from *Rinchenia mongoliensis* ^21^ (* = Oviraptor mongoliensis* ^13^) in their skull morphologies. the anterior margin of the premaxilla is strongly concave above the level at the postodorsal corner of the antorbital fenestra and the highest point of the crest would project far above the orbit in *Corythoraptor jacobsi*, whilst the anterior margin of the premaxilla is nearly straight and the highest point of the crest is above the orbit^2^. There is no pleurocoels on the centra from the second through fourth cervical vertebrae, the anterodorsal margin of ilium (above the acetabulum) is moderately convex and the pubic peduncle of the ilium is larger than the the ischial peduncle in *Corythoraptor jacobsi*, pleurocoels are present on all the cervical centra, the anterodorsal margin of ilium is strongly convex and both peduncles are equal in length in *Rinchenia mongoliensis* ^2^(personal observation).

### Description

The skeleton including skull and lower jaws is almost complete except for the coracoid, and middle-posterior caudal vertebrae (Fig. 1, see also Supplementary Information Table S1). Most of the skull is preserved except for the quadratojugal, a portion of the skull roof and braincase. Some parts of the skull are overlain by the right manus. The skull bears a high crest. Although the anterodorsal part of the crest is missing, its apparent continuous outline implies that the highest point of the crest would project far above the orbit (Fig. 1b, 1c). The internal structure of the crest is similar to the casque of *Casuarius unappendiculatus*. The dorsal part of crest is very thin (about 3.5 mm, measured through its broken surfaces) and was filled with empty spaces. These spaces (pneumatic diverticula) are irregular in shape and differ in size, reminiscent of conditions seen in the modern cassowary. The inner, bony core, casque of the cassowary^25^ is made by irregularly-arranged, slender bony struts called trabeculae^25^. *Corythoraptor jacobsi* possessed an extensive cranial casque that was probably composed of the skull roofing bones: nasals, frontals and parietals (Fig. 2a–c). Only a basal portion, likely a lower half, of the bony core of the casque is preserved and exposed on the left side. The bony core is either obscured by sediment or is exposed due to erosion of the external bony surface. The inner structure is best exposed on the postero-lateral side of the casque. The inner structure consists of randomly branching, sparse, trabeculae of variable thickness ranging from 0.3 (rod-like trabeculae) to 1.2 mm (lamellar trabeculae) (Fig. 2d). These delicate trabeculae outline empty cavities, the largest exposed of which is 6.6 × 14.3 mm large, which implies that the inner core was light, fragile (perhaps pliable), and hence, not suitable for percussive behavior including intraspecific combat. The largest opening (called concavity by Naish and Perron^25^) is in the posterior part of the crest located above the external narial opening (see Supporting Information Fig. S1). The crest extends posteriorly to reach the nuchal margin of the skull. The orbit is circular and probably accommodated a relatively large eyeball. The lower temporal fenestra is rectangular with its long axis vertical. The antorbital fenestra is triangular in lateral view, and larger than the external narial opening. The narial opening is elongate and parallel to the long axis of the antorbital opening.

The premaxillae are completely fused, no sutural remnants are visible. The lower part of the premaxilla is convex outwardly with an almost smooth surface sculptured with irregularly distributed pits. These pits are probably neurovascular foramina, and may indicate the presence of a keratinous sheath (such as rhamphotheca in modern birds) over the premaxillary beak as in ornithomimid dinosaurs^26, 27^. The ventral margin of the premaxilla is sharp and forms a crenulated tomial edge. The broken surface of the ventral margin of the premaxilla shows that the bone consists of many chambers separated by thin bony struts, and is thus largely pneumatized and lightweight. Anterior to the ventral corner of the external narial opening, the lateral surface of the premaxilla is moderately concave. The supranarial (nasal) and subnarial (maxillary) processes of the premaxilla^2^ contact the nasal as in other derived oviraptorids. The subnarial process of the premaxilla is slightly convex with a smooth surface, strap-like, and extends posterodorsally at about 40 degrees with the ventral margin of the skull. This process forms the ventral margin of the external narial opening. Unlike other oviraptorids, the supranarial process of the premaxilla bears two processes: a short posterodorsally extending process, forming the anterodorsal margin of the external nasal opening, and a long process, forming most of the anterodorsal process of the premaxilla. There is a distinct ridge on the lateral surface near the anterior margin of the premaxilla. The external narial opening is elongate and located above the antorbital fenestra. The long axis of the narial opening is parallel to the long axis of the antorbital fenestra. In ventral view, the fused premaxillae are U-shaped, with three longitudinal ridges on the ventral surface. The middle ridge is much thicker than the lateral and medial ones. Two deep grooves are developed between the ridges. A large fossa is present on the ventral surface of the premaxillae near the premaxillary-maxillary suture.

The short maxilla contacts the premaxilla anteriorly. The lateral margin of the maxilla is missing. The maxilla bears a slender process closely appressed to the subnarial (maxillary) process of the premaxilla, which extends posterodorsally. This process ends before reaching the posterodorsal corner of the antorbital fenestra. Thus, the anterior corner of the antorbital fenestra is demarcated by the maxilla. Three small openings are present on the lateral surface of the maxilla near the anteroventral corner of the antorbital fenestra. The one nearest the anterior margin of the antorbital opening is a maxillary fenestra, and other two are pneumatopores as in other derived oviraptorids such as *Citipati* and *Conchoraptor* ^2^. The lateroventral surface of the maxilla is convex and smooth. It extends ventrally toward the mid-line near the suture with the premaxilla, forming a distinct tooth-like process as in other oviraptorids.

The anterior margin of the lacrimal is straight. The broken surface shows heavily pneumatized internal bone.

The ascending process of the jugal is curved posteriorly in lateral view. The ascending process of the jugal occupies about two-thirds of the length of the postorbital bar.

The postorbital is T-shaped with anterior, posterior, and ventral processes. The posterior process extends posteromedially and covers the anterior part of the squamosal. With the anterior process, the postorbital forms the lateral margin of the supratemporal fenestra. The elongate supratemporal fenestra is much smaller than the orbit and the infratemporal fenestra. The ventral process of the postorbital is relatively long, but it cannot be determined whether it reaches the posteroventral corner of the circular orbit due to its incomplete distal end. The preserved portion of the ventral process occupies at least two-thirds the length of the postorbital bar. In lateral view, the posterior margin of the ventral process is straight and forms the anterior margin of the infratemporal fenestra.

The preserved portion of the nasals exhibits highly pneumatized bone structure. The suture between the parietal and frontal is not clear, but it seems the bones project dorsally and formed a distinct crest together with the nasals.

The occipital condyle is preserved and extends posteroventrally. A large opening on the lateral surface on the occipital condyle is probably for cranial nerve XII.

The mandible is toothless. The lateral surface of the dentary is slightly concave and covered with foramina. The symphyseal suture is straight. The mandibular symphysis is U-shaped in dorsal view. The rostral end of the mandibular symphysis is slightly downturned in lateral view. The external mandibular fenestra is large, longer anteroposteriorly than dorsoventrally. The cranial margin of the fenestra is deeply incised and divides the dentary into two long, shallow dorsal and ventral caudal processes. The angular and surangular bones are separated by a gap and the posterior rim of the external mandibular fenestra. The dentary is W-shaped in ventral view because of a distinct process at the ventral end of the mandibular symphysis with large concavities on each side. This concavity may have accommodated the splenial.

The cervical series is complete, and almost naturally articulated. The circular curl of the neck is similar to that of *Heyuannia* ^22, 28^, which doesn’t seem like the typical death pose of theropods, although what caused this is unclear. Including the atlas, there are twelve cervical vertebrae (Fig. 1d). The sixth and eleventh cervical vertebrae are the longest among the series. There is no pleurocoel on the second through fourth cervical vertebrae, but one is present in the fifth through twelfth cervical vertebrae. The pleurocoel is nearly circular on the fifth cervical vertebra (about 4.8 mm in diameter). It is oval in the sixth cervical vertebra (about 5 mm long). All pleurocoels are located in the middle of the centra. The pleurocoels have sharp lower margins but their upper boundaries are less distinct. The anterior articular surfaces of centra are strongly concave and the posterior articular surface becomes moderately convex and oblique anteroventrally to posterodorsally. The anterior articular surfaces are almost square and wider than the posterior articular surfaces. The widest part is situated between the parapophyses. The cervical ribs are fused with the vertebrae and bear distinct anterior processes. The weathered surface of neural arch shows many small chambers separated by thin bony struts within the neural arch, which indicate that the neural arches are densely pneumatized. The exposed neural spine is low and triangular in lateral view. A centrodiapophyseal lamina is well-developed on the fourth to seventh cervical vertebrae. A distinct concavity lies above the centrodiapophyseal lamina and below the postzygodiapophyseal lamina at their junction.

Only the first six dorsal vertebrae are exposed, thus the total number of the dorsal vertebrae is not determined. The dorsal vertebrae are shorter in length than the cervicals. The first hypapophysis is a distinctive plate-like projection, which extends anteroventrally. The hypapophyseal body is anteriorly placed, but its posterior extension reaches almost to the posterior edge of the centrum. Its distal end is rounded. Part of the second dorsal vertebra is covered by a dorsal rib. The hypapophysis of the third dorsal vertebra is also distinct, similar to that of the first dorsal vertebra. The second and third dorsal vertebrae bear larger pleurocoels than those of cervical vertebrae. The anterior surfaces of the dorsal vertebrae are slightly concave and their posterior articular surfaces are nearly flat.

Only the posterior two sacral vertebrae are observable and they have a small pleurocoel. The rib of the last sacral vertebra is stout and contacts the postacetabular process of the ilium medially. Both the ventral and lateral surfaces of the sacral centra are smooth and round.

The anterior five caudal vertebrae are preserved, and the first three are complete. The first caudal vertebra abuts the posterior sacral vertebra. Both the anterior and posterior articular surfaces of caudal vertebrae are nearly flat. The anterior articular surface is circular in cranial view. The pleurocoel is small and elongate on the first and second caudal vertebrae. The pleurocoels of the third through fifth caudal vertebrae are somewhat larger, but do not match the size of those of the dorsal vertebrae. The transverse process of the first caudal vertebra is long and projects posterolaterally. There is no infraprezygapophyseal fossa on the first caudal vertebra. There are three fossae on the other caudal vertebra as in *Nankangia* ^6^: the largest fossa (called infraprezygapophyseal fossa) is located on the anterior surface near the junction of the prezygapophysis and the transverse process; the second fossa (called the infradiapophyseal fossa) is exposed ventral to the base of the transverse process; and the third fossa is the pleurocoel. The neural arches of the caudal vertebrae are similar to those of *Nankangia* ^6^. Only a small proximal portion of haemal arch, which starts between the second and third caudal vertebrae, is preserved.

Only a thin strap-like distal end of the scapula is exposed. The very thin sternum is present, but not well preserved.

The left humerus is almost complete except for its broken proximal portion. The humerus is approximately 27% of the entire forelimb length including the manus and 48% of the forearm length without the hand (Supplementary Information Table S1), and is weakly twisted as in other oviraptorids. The deltopectoral crest is short and located at the proximal portion of the humerus (ratio of deltopectral crest to total length of humerus, measured along the humeral shaft, = 31%). The distal humerus is expanded and bears well-developed rounded condyles. Both lateral condyles and the medial epicondyle are broken, but the former seems larger than the latter. The lateral epicondyle is well developed. In anterior view, a distinct concave surface is visible between the lateral condyle and epicondyle.

The ulna is slightly shorter than the humerus. It is short, approximately 26% of the length of the forelimb including the manus, bowed and convex caudally with a poorly developed olecranon. The proximal end is more expanded than its distal. The medial surface of the distal portion is nearly flat.

The radius is rod-like and slightly shorter than the ulna. It is moderately curved cranially, thus leaving a space between the ulna and radius as in *Heyuannia* ^22, 28^. The radial shaft exhibits a uniform width along its length, but is much narrower than the ulna. The radius has an expanded distal end.

The proximal ends of metacarpals are closely appressed. The first metacarpal is the shortest. It is slightly stouter and about 41% the length of the second metacarpal. The ventral surface of the first metacarpal is slightly concave. Metacarpal II is moderately robust, with a shaft diameter about 13% of total length. The second metacarpal has a circular shaft. The distal articular surface of the second metacarpal has a well-developed trochlea. The medial condyle is more developed and extends more medioventrally than the lateral condyle. In ventral view, the shafts of the second and third metacarpals are convex medially, thus leaving a gap between the distal ends of the second and third metacarpals. The third metacarpal is narrower than the second; the former is about 68% of the width of the latter. Its proximal portion is expanded dorsoventrally rather than mediolaterally. The middle shaft of metacarpal III is slender, the diameter being about 9% of the bone length. Metacarpal II and III are equal in length.

The manual phalanges are long and robust. Manual phalanx I-1 is stout and the longest among the phalanges, being about 72% of the length of the metacarpal II, and strongly constructed, with diameter about 15% of its length. A large collateral ligament pit occurs laterally and medially.

The distal portion of left manual ungual I is missing. This laterally compressed ungual is the largest of the manual unguals. The weakly curved claw has a simple, single groove on its lateral and medial surfaces, with the medial groove being deeper. The flexor tubercle is developed as a large, mound-like rugosity. The proximal articular surface extends dorsally and forms a well-developed tongue-like projection. Manual phalanges (MP) II-1 and II-2 are long and moderately robust, each being about 67% and 66% of length of the metacarpal II, respectively. The collateral ligament pits are strongly developed. The proximal articular surface of MP II-2 is concave with a moderate-sized articular heel and tongue. The second manual ungual is smaller, proportionately more elongate, and more weakly curved than the first manual ungual. Its proximodorsal lip is similar to that of the first manual ungual, but the flexor tubercle is smaller. Manual phalanges III-1 and III-2 are shorter than MP III-3. MP III-2 is the shortest among the phalanges. The third manual ungual resembles the ungual of digit II in shape, but is slightly smaller than the unguals of digit I and digit II. The ungual of digit III is not as strongly curved as other unguals. Its proximodorsal lip is developed, as in other unguals.

Both ilia are preserved, but the postacetabular process of the right ilium is incomplete. The postacetabular and the preacetabular processes of the left ilium are missing their distal ends, but the impression of the preacetabular process and the outline of the postacetabular process provide a precise shape and size of the ilium. The dorsal margins of both iliac blades are close to each other medially. The distal ends of the postacetabular processes are more greatly separated than the preacetabular processes. The preacetabular and postacetabular processes are similar in length. The lateral surface of the postacetabular process is convex, and the middle portion of the ilium above the acetabulum is slightly concave. A distinct brevis fossa is present on the ventral surface of the distal end of the postacetabular process. The hook-like distal end of the preacetabular process extends below the level of the dorsal margin of the acetabulum. The pubic peduncle is deeper than the ischial peduncle. A distinct process occurs on the posterior margin near the base of the pubic peduncle. Anterior to the pubic peduncle, a shallow elongate cuppedicus fossa is situated at the ventral surface of the preacetabular process. The brevis fossa, located on the ventral surface of the distal end of the ischial peduncle, is shallow and short. A weak antitrochanter occurs on the ischial peduncle. The pubic peduncle is deeper dorsoventrally and narrower anteroposteriorly than that of the ischial peduncle.

Both pubes are well preserved and naturally articulated with ilium and ischium. As in most other derived oviraptorids, the pubis is concave cranially, but differs from *Nomingia*, where the pubic shaft is almost staight^3^. The shaft is mediolaterally compressed distally and becomes more rounded posteriorly near its middle portion but becomes mediolaterally compressed again toward to the proximal end. A pubic apron extends medially from the proximal end along the middle margin of the shaft, although some parts are broken. It occupies the 53% the whole length of the pubis. Distally, pubic aprons are completely fused to form a symphysis (pubic boot). The pubic boot has a well-developed cranial process and a caudal process. The cranial process (7 cm long) is longer than the caudal process (5 cm long), which is different from that of *Nomingia*, which bears an equally long processes^3^.

Both ischia are preserved, but are partially overlain by the right pes. As in other oviraptorids, the ischium has a medially positioned triangular obturator process. The obturator process is located approximately midway along the shaft. Its lateral surface is concave with the shaft mediolaterally compressed and concave caudally. The distal ends of both ischia are not fused.

The femur is longer than the ilium as in oviraptorids and forms about 30% of the total hindlimb length. It is straight in both lateral and posterior views. The femoral neck is directed dorsomedially at an angle of about 115° to the shaft. A distinct short ridge-like structure occurs on the posterior surface of the femoral head. The greater trochanter is massive and extends craniocaudally. It is separated from the femoral head by a slightly constricted femoral neck. There is no sign of lesser trochanter (cranial trochanter), which may be fused into the greater trochanter. There is no distinct fourth trochanter, but a muscular scar lies along the shaft in the region where a fourth trochanter would be expected to reside. This coarse surface (41 mm long and 15 mm wide) is also present in *Citipati osmolskae* and *Khaan mckennai* ^29^. The dorsal surface of the greater trochanter is 113 mm from the middle of the coarse area. The distal end is of the femur broad with a nearly flat cranial surface. On the posterior surface, the distal femoral condyles are well separated, with the lateral condyle projecting well below the medial one. A deep popliteal fossa is present between the two distal condyles. Caudally, a shallow fossa separates a well-developed tibiofibular crest from the large caudal surface of the medial condyle on the posterolateral surface of the distal end of the femur. An extensive tibiofibular crest projects posteriorly beyond the level of the medial condyle.

The tibia is 19% longer than the femur. In medial view, the dorsal margin of the proximal end is convex. The pronounced cnemial crest is inflected anteriorly and slightly medially. It constitutes about half of the entire craniocaudal length of the proximal surface of the tibia. There is no distinct boss on the distal end of the crest, similar to the condition in *Khaan mckennai* ^29^. The cnemial crest of the tibia is 52.9 mm long and it extends to approximately the proximal 14% of the tibia, and ends at the proximal margin of the fibular crest. The proximal portion of the fibular crest is ridge-like, extending posterolaterally, ending 130 mm from the dorsal margin of the proximal end of the tibia. Lateral to the distal end of the fibular crest, there is a small foramen with a short dorsal groove associated it. This foramen is likely a nutrient foramen, which is similar to that of *Khaan mckennai* (IGM 100/973)^29^. The distal tibiae are not well preserved but appear more flattened anteroposteriorly. Distal ends of the tibiae are not well-preserved.

The distal portion of the fibula is missing. The proximal head of the fibula expands anteroposteriorly. The fibular head is weakly concave medially as in most oviraptorosaurs^2^.

Only a part of the astragalus, which is articulated with the tibia, is preserved. The calcaneum is damaged, thus detailed description of the astragalus and the calcaneum is not available.

The pedes are exposed ventrally. The foot accounts for approximately 29% of the length of the hindlimb. The proximal portion of the left foot is missing. Two separate distal tarsals were found; the lateral distal tarsal IV is larger than the medial distal tarsal III. The lateral tarsal covers the proximal articular surfaces of the metatarsal II and III. The medial metatarsal covers the proximal articular surface of the metatarsal IV. Each tarsal fuses with the corresponding metatarsal, which is similar to those of some caenagnathids (*Elmisaurus*, *Leptorhynchos*)^30, 31^. Metatarsals I through V are preserved. The stout metatarsal III is longest. It is exposed along its entire length and exhibits almost constant width. Metatarsal IV is slightly shorter than metatarsal III but longer than metatarsal II. Metatarsal V is a thin splint of bone; its distal portion is missing. Deep ligament foveae are present on medial and lateral surfaces of the distal ends of the metatarsals II-IV. The foveae are much larger and deeper on metatarsal III than others. In ventral view, 56.6 mm away from the distal ends of metatarsals II-IV, a coarse area occurs on each metatarsal. The proximal ends of metatarsals III and V expand mediolaterally and are wider than that the proximal end of the metatarsal II, which expands anteroposteriorly. The distal ends of the metatarsals bear distinct trochleae, with medial margins stronger than lateral. The distal ends of metatarsals II-IV are twisted medially; the distal end of the metatarsal II is not prominent.

*Corythoraptor jacobsi* has the typical theropod phalangeal formula of 2-3-4-5. The left pes is preserved in a natural pose, exposing its ventral surface. Digit 3 is longest. Digit 4 is longer than the digit 2. Digit 1 is shortest, being 2/3 the length of the first phalanx of the digit 2. All phalanges possess a deep ligament fovea on the lateral and medial surfaces. These pits are circular in shape. The phalangeal joints are symmetrical and ginglymoid. The unguals are moderately curved and bear a shallow groove running along the medial and lateral surfaces; the groove becomes shallower more distally. A long tongue-like dorsal lip occurs near the proximal end.

### Phylogenetic analysis of *Corythoraptor jacobsi*

**Figure 3:**
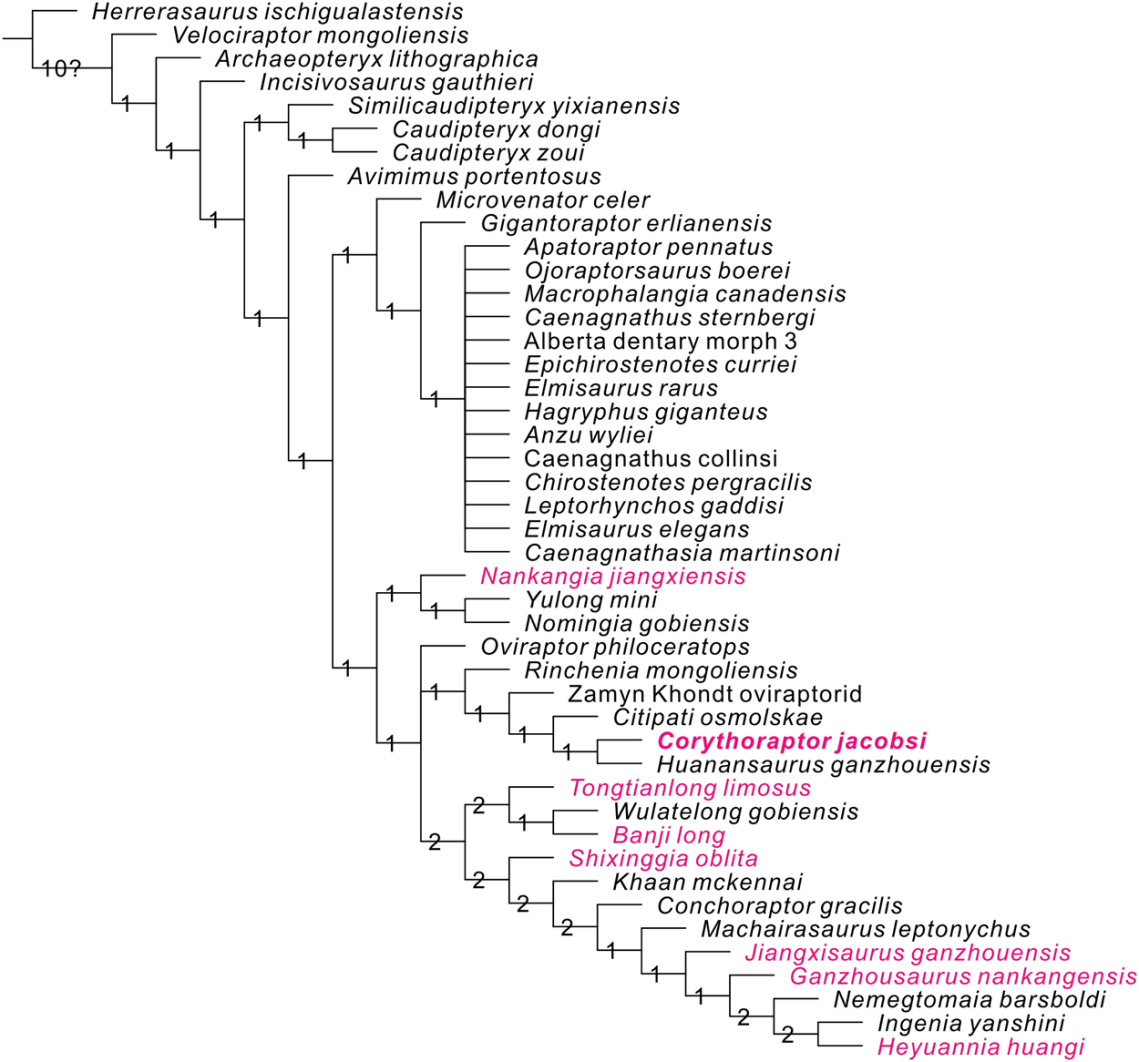
Strict consensus of 4151738 most parsimonious trees obtained by TNT, based on analysis of 45 taxa and 257 characters, showing the phylogenetic position of *Corythoraptor jacobsi* gen. et sp. nov. (Tree length = 623). Numbers adjacent to each node are Bremer support values. Oviraptors from southern China are in red.

The strict consensus tree (Fig. 3) shows all oviraptorid dinosaurs from southern China are mainly distributed in three main clades of Oviraptoridae. *Corythoraptor jacobsi* and *Huanansaurus ganzhouensis* form one clade, and they share the following seven synapomorphies: 1) posterodorsally inclined postorbital process of the jugal (character 37, state 0); 2) dentary, anterodorsal tip of beak: projecting anterodorsally, tip of beak projecting at an angle of 45° or less relative to the ventral margin of the symphysis (character 191, state 1); 3) pneumatized dentaries (character 199, state 1); 4) posteroventral branch of dentary twisted so that lateral surface of branch faces somewhat ventrally (character 226, state 1); 5) development of symphyseal shelf of mandible: length of symphysis (as measured on midline) greater than 20% but less than 25% length of mandible (character 229, state 1); 6) prominent proximodorsal extensor ‘lip’ on manual unguals (‘set off’ from remainder of dorsal surface by distinct change in slope immediately distal to ‘lip’) (character 234, state 1); and 7) external mandibular fenestra: anteriorly constricted by posteroventral ramus of dentary (character 254, state 1).

The strict consensus tree also recovers that oviraptorid dinosaurs from Ganzhou area are nested in three subclades of Oviraptoridae, and better resolved than the tree obtained by Lü *et al*.^9^. It is worth mentioning that *Nangkangia*, *Yulong* and *Nomingia* form a clde. However, *Nankangia* and *Yulong* were regarded as being plant-eating^6, 17^, thus, these oviraptorid dinosaurs nested in different clades may imply different ecological niches.

### Bone microstructures of *Corythoraptor jacobsi*

Three samples from rib, right fibula and left radius of the holotype of *Corythoraptor jacobsi* for histological study were acquired (see also Supplementary Information). The preserved fibular histology exhibits intensive bone remodeling that removed early growth marks. The remaining fibular growth marks would suggest that the holotype of *Corythoraptor jacobsi* was an individual with an age of more than 6 years.

**Figure 4:**
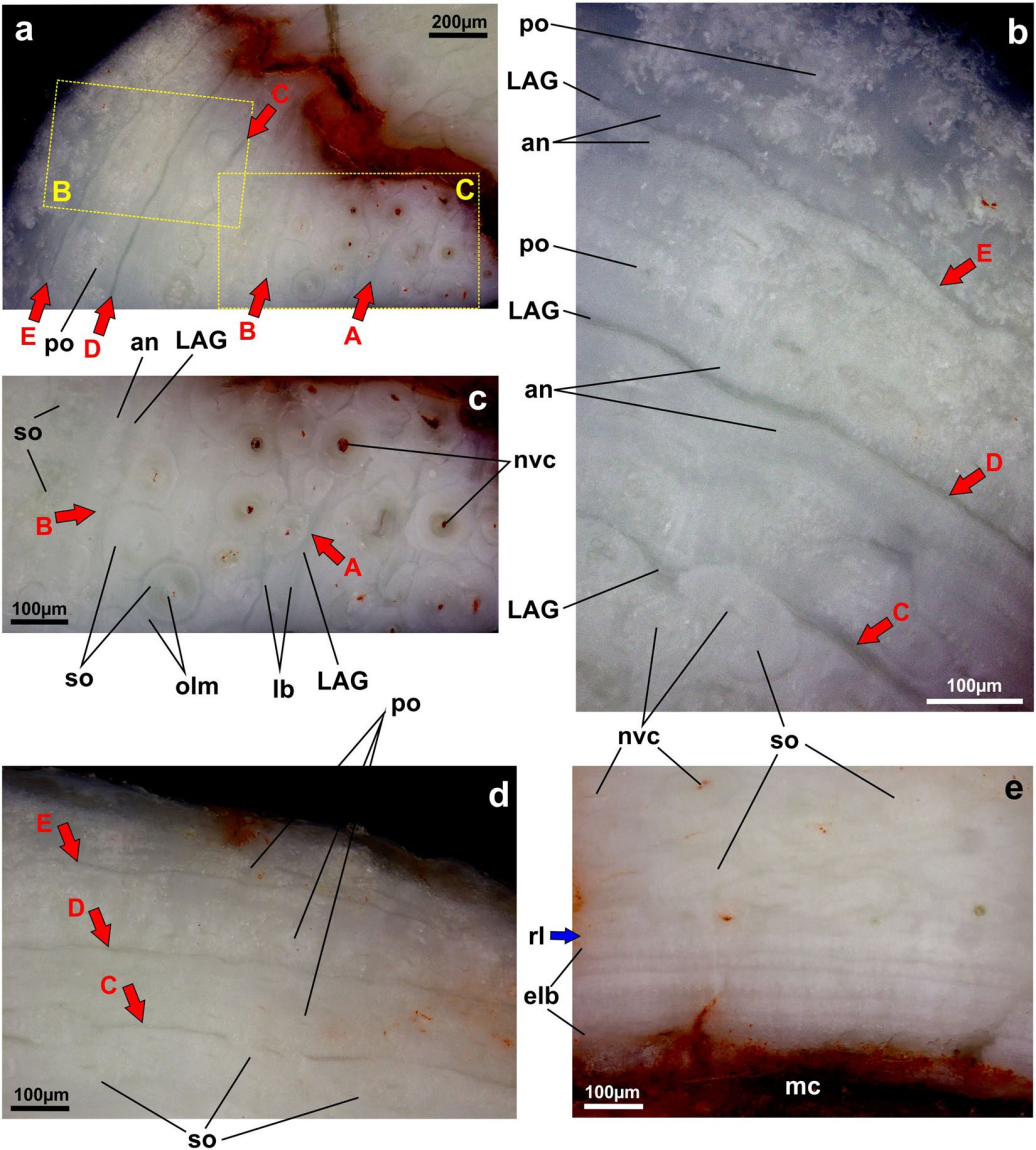
Radius microstructure of the holotype of *Corythoraptor jacobsi* gen. et sp. nov. (JPM-2015-001) viewed with reflected light microscopy. (**a**) Transversal section of the midshaft showing five growth lines (red arrows A to E) present in the middle-to-outer cortex. Note that growth line spacing decreases periosteally. (**b**) Close-up of the outer cortex with three growth lines (C to E). Note avascular layers (annuli) deposited prior to and after darker undulating line (line of arrested development, LAG). Primary bone is mostly preserved external to the growth line D. Secondary osteons partly obliterate the growth line C. (**c**) Close-up of the middle cortex with two growth lines (A and B). Growth line A consists of an ill-defined LAG followed by laminar bone (annulus). Growth line B comprises a darker line (LAG) and lighter avascular layer (annulus). Note several generations of overlapping secondary osteons are present. (**d**) The last growth cycles delimited by growth lines C through E. (**e**) Interior margin of the cortical bone with a thick deposition of endosteal laminar bone. The blue arrow points to the resorptive margin. Abbreviations: an, annulus; elb, endosteal laminar bone; LAG, line of arrested growth; lb, laminar bone; mc, medullary cavity; nvc, neurovascular canal; osla, osteonal laminae; po, primary canal; rl, resorption line; so, secondary osteon.

Radius (Fig. 4, Supplementary Information Fig. S4) – Bone microstructure of the radius midshaft has provided valuable information about the growth stage and helped to estimate the histological age of the type specimen of *Corythoraptor jacobsi*. The cortex is composed of both primary and secondary bone tissue; the latter being dominant throughout the section. The cortex is interrupted by five (A to E revealed in reflected light; Fig. 4a) or six growth lines (A to F revealed in polarized light; Supplementary Information Fig. S4a), although more may have been lost to extensive remodeling and erosion. The primary bone is formed by fibro-lamellar tissue (indicating rapid osteogenesis) with osteons typically arranged in a laminar pattern. Osteonal canals are oriented longitudinally. This tissue remained mostly external to the growth line D (Fig. 4b).

The growth lines are composed of the dark component referred herein to the line of arrested growth (LAG) and two adjacent brighter avascular layers considered to represent the annulus. Only growth line A is slightly modified, having a band of several distinct lamellae deposited after LAG (Fig. 4c). The three-component growth lines indicate that during seasonal retardation of growth, rapid osteogenesis slowed down (to form pre-annulus), and after temporary interruption of bone deposition (expressed by LAG) osteogenesis resumed at slower rates first (to form post-annulus). Annular depositions are variably thick. Pre-annulus of the growth line D and E is 47 µm and 27 µm wide, respectively, whereas corresponding post-annuli are 23 µm and 28 µm wide (Fig. 4b). However, pre-annulus of growth line A is only 13 µm wide while post-annulus (lamellar bone) is much wider (49 µm, Fig. 4c).

In contrast to the fibula, the spacing between concentric growth lines in the radius diminishes moderately towards the periphery according to zonal measurements collected in reflected light: AB-zone = 357 µm; BC-zone: 242 µm, CD-zone: 187 µm, DE-zone 180 µm, and E-periosteal surface zone (lacking any growth mark near external periphery) 213 µm (Fig. 4a). The fastest recorded radial growth rate of 0.96 µm is calculated for a 371-day-long Cretaceous year^32^. However, daily bone deposition rate might be higher earlier in life and its estimation also depends on how long the rapid growth season lasted in each year. Zonal measurements collected from another portion of the cortex in polarized light (Supplementary Information Fig. S4a) are slightly different but correspond to gradual slowing of somatic growth: AB-zone: 145 µm, BC-zone: 284 µm, CD-zone: 136 µm, DE-zone: 125 µm, EF- 117 µm. These measurements imply that the minimum age estimated based on preserved histology might be around seven years.

Most peripheral secondary osteons occur within the CD-zone (Fig. 4c,d; Supplementary Information Fig. S4b). Internal to growth line B, the Haversian bone tissue is dense due to repeated remodeling that makes it impossible to discern tissue discontinuities referable to early growth marks. Secondary osteons are variable in size, ranging from 100 × 109 µm to 131 × 137 µm. The earliest growth marks likely disappeared by medullary cavity expansion that considerably eroded the innermost cortical surface.

The cortex encloses a large medullary cavity devoid of cancellous bone (Fig. 4e). Several deposition cycles of endosteal bone line the entire margin of the medullary cavity (Fig. 4e, Supplementary Information Fig. S4c). This internal circumferential layer is composed of avascular parallel-fibered bone and is variable in thickness (193 to 292 µm). It is up to almost seven times thicker than the fibular endosteal bone.

The animal perished at the beginning of a new season as is exemplified by resumed bone deposition nearest to the periosteal surface. We suggest that the holotype of *Corythoraptor jacobsi* had not reached maximum body size and was still growing at the time of death. The ontogenetic stage of the holotype individual probably corresponds to a young adult that was approaching a stationary stage of development^33^. It is reasonable to assume that the prominent casque served as a sexual signaling ornament, what would imply that these oviraptorids were reproductive before they finished growing. Finally, the osteochronology exhibits that *Corythoraptor jacobsi* required more than 8 years to reach somatic maturity.

## Discussion

### Putative functions of the cassowary-like crest of *Corythoraptor*

Recent cassowaries, the flightless birds from New Guinea, Australia and the Aru archipelago^34^, also evolved cranial casques of diverse shapes (Fig. 2e, 2f). However, the bony core of the cassowary casque incorporate much denser web of microtrabeculae that is able to deform when subjected to pressure^35, 36^. Many of the trabecuale are rod-like with diameter about 0.1 mm (Fig. 2g), whereas trabeculae situated close to peripheries of the casque are lamellar or plate-like measuring across up to 3 mm (Fig. 2h). The empty spaces are considerably smaller in cassowaries and bony shell of the casque is sheated by keratin (Fig. 2i). Rugose external surface suggests that cranial casque was likely covered by outer keratinous sheath in *Corythoraptor* as well. The shell-like outer layer of the casque is approximately 2-3 mm thick in the cassowary^25^ and 2 mm thick in *Corythoraptor*.

These peculiar morphological similarities make the cassowaries the closest living analogue that provides some clues for assuming putative functions of the casque in the oviraptorid *Corythoraptor*. Based on comparisons with the cassowary model, we put forward three hypotheses to explain plausible functions of the *Corythoraptor* casque: 1) termoregulatory hypothesis – larger inner cavities overlying could effectively dissipate heat produced inside of the braincase; the function demonstrated for the casque of the cassowary^37^; 2) acoustic hypothesis – Kundrát and Janáček^38^ suggested the supraencephalic pneumatic pathway connecting contralateral middle ear cavities indicate enhancements of acoustic perception in the lower-frequency registers in the oviraptorid *Conchoraptor*. The casque microstructure and positional proximity to tympanic recesses enabled the *Corythoraptor*´s helmet could function as a resonator amplifying or/and pointing low frequency signals over a much greater range. Increased perception could be then utilized for predator avoidance or prey capture. and 3) sociosexual hypothesis partly overlap with the acousting hypothesis as more efficient low-frequency communication linked to more strongly resonating casque evolved under sexual selection. Recent cassowaries produce low-frequency casque-directed vocalizations towards a partner during mating season^25, 39^. Low-frequency sounds, however, are made inside the throat apparatus in the cassowaries^25^ and there is no morphological evidence for such behavior in oviraptorids. Moreover, this behaviour would require a casque structure to evolve in both sexes of the same species at least. Thus there is at the moment a little support for the *Corythoraptor*´s casque was used solely for acoustic-sexual signalling. Larger and probably more ornamented casque rather provided members of one or both sexes with display structure of hierarchic intraspecific status. However, we have to stress here the fact, the male and female cassowaries exhibit similarly ornamented with large casque^25^.

**Figure 5:**
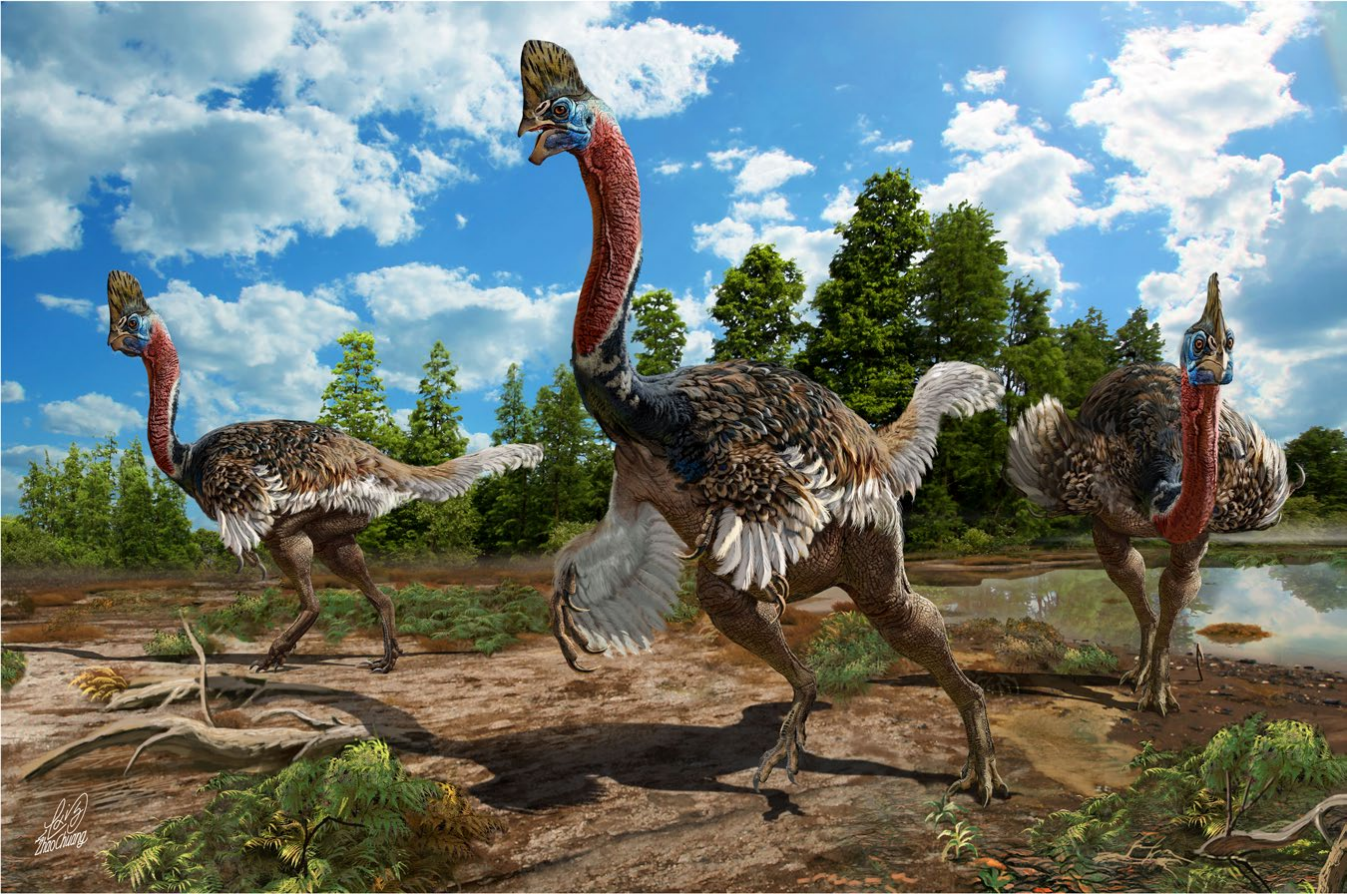
The living scene of *Corythoraptor jacobsi* gen. et sp. nov. (Drawn by Zhao Chuang).

The cassowary-like crest in the skull is similar to the casque of cassowaries (Fig. 5), which serves a sociosexual role and functions in visual and acoustic display^25^. It is therefore reasonable to assume that the cassowary-like crest of *Corythoraptor jacobsi* was probably utilized in a similar way. The sharp claw and long neck of *Corythoraptor jacobsi* may also indicate that its living behavior is perhaps similar to the modern flightless cassowary. It seems more reasonable to assume that the cassowary-like crest of *Corythoraptor* was more likely the multifunctional structure that conspicuously expressed fitness, and probably sex, of each individual of *Corythoraptor*.

The histological study revealed that the type specimen of *Corythoraptor jacobsi* was probably at least eight years old but still not a fully grown individual. Finally, the discovery of *Corythoraptor jacobsi*, the seventh oviraptosaurian taxon from the region, provides evidence of unprecedented morphological and taxonomic diversity of this clade in the Ganzhou area, China.

*Corythoraptor jacobsi* gen. et sp. nov. represents the first oviraptorid dinosaur with a highly developed cassowary-like skull crest from China. Phylogenetic analysis indicates that *Corythoraptor jacobsi* belongs to the clade of Oviraptoridae, and shows a close relationship with *Huanansaurus*.

## Methods

### Phylogenetic analysis

We conducted a phylogenetic analysis to investigate the taxonomic affinities of *Corythoraptor* within Oviraptorosauria, using the modified data matrix^9, 40^ (see also Supplementary Information) of Lamanna *et al*.^41^. With the addition of *Corythoraptor* into the modified data matrix, 45 taxa (*Herrerasaurus*, *Velociraptor* and *Archaeopteryx* as outgroups; 42 taxa as ingroups) and 257 osteological characters was analyzed using TNT (Tree Analysis Using New Technology) version 1.1 (Willi Hennig Society Edition)^42^. A traditional search (tree bisection-reconnection swapping algorithm, 1,000 random seeds, 1,000 replicates, 10 trees saved per replication) yielded 4151738 most parsimonious trees with 623 steps.

### Analysis of the bone mircostructures

Three samples for histological study were acquired as small fragments extracted from rib, right fibula and left radius of the holotype of *Corythoraptor jacobsi*. The samples were taken from near midshaft of the bones. Thin sections of the samples revealed that original histostructure was petrographically altered and is barely visible in transmitted light (Supplementary Information Fig. S2A). Some histological information, however, became accessible when we investigated polished bone samples using reflected light microscopy (e.g., Supplementary Information Fig. S2B–D), as well as we examined the thin sections with circular polarized light (e.g., compare Supplementary Information Fig. S3A,B).

The petrographic thin sections were prepared according to the following methodology: 1) bone samples were embedded in bicomponent epoxy resin (Lamit 109; Kittfort); 2) the embedded samples were ground on a Montasupal grinder (Germany) using SiC (grain size: 400–600 nm); 3) warm re-impregnation of the ground surface with EpoFix (Struers); 4) fixation of the samples to slides using epoxy resin (type 109); 5) fixed samples were sectioned using a diamond knife (diameter 150 mm, Struers); 6) fixed samples were thinned on the Montasupal grinder using the abrasives of 240, 400 and 600 grits combined with ultrasound cleaning to reach a thickness of 0.2 mm; 7) final manual abrasion using 1000 grit SiC to reach a thickness of 30 microns; and finally 8) the sections were cover-slipped using a synthetic resin or polished on the Planopol TS (Struers). Preparation of polished bone samples followed the above steps: 1 through 3 and 5 through 7.

The cover-slipped sections and polished samples were examined using crossed polarized light and reflected light microscopy. Photography was carried out using digital camera Olympus XC50 (operating software: Olympus Stream Start; Olympus Soft Imaging Solutions GmbH) mounted on the Olympus BX51 microscope. The images were processed using Adobe Photoshop and CorelDRAW X5 software. Histological measurements were taken from digitized cross sections using ImageJ.

### Data archiving

Specimen measurements, the phylogenetic character scores for *Corythoraptor*, and the phylogenetic topology with synapomorphies are available as Supplementary Information.

## Electronic supplementary material

Supplementary Information 
