# Supplementary material for: High diversity of the Ganzhou Oviraptorid Fauna increased by a new “cassowary-like” crested species
Source: Sci Rep. 2017 Jul 27;7:6393. doi: 10.1038/s41598-017-05016-6 (PMC5532250; doi:10.1038/s41598-017-05016-6)
Supplement: Supplementary file 1 — Supplementary Information [file 41598_2017_5016_MOESM1_ESM.pdf]

Supplemental information for:

High diversity of the Ganzhou Oviraptorid Fauna increased by a new "cassowary-like" crested species

Junchang Lü[1*], Guoqing Li[2], Martin Kundrát[3], Yuong-Nam Lee[4], Zhenyuan Sun[5], Yoshitsugu Kobayashi[6], Caizhi Shen[1], Fangfang Teng[7], Hanfeng Liu [2]

1. Institute of Geology, Chinese Academy of Geological Sciences, Beijing100037, China; Key Lab of Stratigraphy and Paleontology, Ministry of Land and Resources of China, Beijing 100037, China, Lujc2008@126.com
2. Jiangxi College of Applied Technology, Ganzhou, 341000, Jiangxi Province, China
3. Center for Interdisciplinary Biosciences, Faculty of Science, University of Pavol Jozef Safarik, Kosice 04154, Slovak Republic
4. School of Earth and Environmental Sciences, Seoul National University, Seoul 08826, South Korea
5. Jinzhou Paleontological Museum, Jinzhou 121000, Liaoning Province, China
6. Hokkaido University Museum, Hokkaido University, Sapporo, Hokkaido, 060-0810, Japan
7. Xinghai Paleontological Museum of Dalian, Dalian 116000, Liaoning Province, China

Contents

1. Table S1
2. Supplementary Fig. 1.
3. Bone microstructures
4. Character list and datamatrix for phylogenetic analysis
5. Supplementary Fig. 5.
6. References

1. Table S1. Measurements (in mm) of *Corythoraptor jacobsi* gen. et sp. nov. (JPM-2015-001)

	L	W (H)
preorbital skull length	88.8	
Basal skull length	174.4 (est.)	
Across premaxilla–maxilla suture of the snout	43.1	41.1
The tomial margin of the premaxilla	36.3	31.3
Maxilla (lateral view)	47.1	-
Orbit	50.9	52.4
Antorbital fossa	41.8	-
Dentary	77.4	48.2
External mandibular fenestra	43.9	32.6
Lower jaw	143.2(e)	-
2nd to 8th cervical vertebrae	52 (without ont); 55.3;67.8;66.4;71.7;62.6; 63.7	
9th to 12rd cervical vertebrae	64.5;65.7;71.4;53.2	
Whole cervical series	800	-
Whole dorsal series	400	-
Sacral series	210	-

Humerus	212.9	22.9
Deltopectoral crest	66.2	-
Ulna	210.0	11.7
Radius	205.0	11.0
Metacarpal I	39.4	14.6
Manual phalanges I-1,2	69.5; 60.5(est.)	10.4;28.7
Metacarpal II	96.5	12.7
Manual phalanges II-1,2,3	64.5;63.5;60.9	10.2;7.7;24.4
Metacarpal III	93.8	8.6
Manual phalanges III-1,2,3,4	36.3;31.1;50.9;55.6	10.0;10.0;8.4;22.6
Entire forelimb length including the manus	790	
Entire forelimb length without the hand	440	
Ilium	300	110
Acetabulum	51.1	-
Preacetabular process	149.6	90.3
Postacetabular process	150.4	79.6
Ischium	220	26
Pubis	360	22
Pubic apron	190	-
Femur	330.5	33
Tibia	392	28.9
Metatarsals II-V	148.3;157;156;45	12.6;15.1;15.9
Digit 1-1,2	37.2;36.9	-,-
Digit 2-1,2,3	49.2;30.4;52.1	11.7;11.6;9.3
Digit 3-1,2,3,4	48;30.5;30.5;54.2	13.6;12.6;10.0;8.7
Digit 4-1,2,3,4,5	33.2;24.4;22.9;28.9;49.6	14.2;14.1;12.8;10.4;7.0

Hindlimb length with pes	1090	-
Hindlimb length without pes	730	-
Pes (without metatarsal)	315	

2. Supplementary Fig. s1.

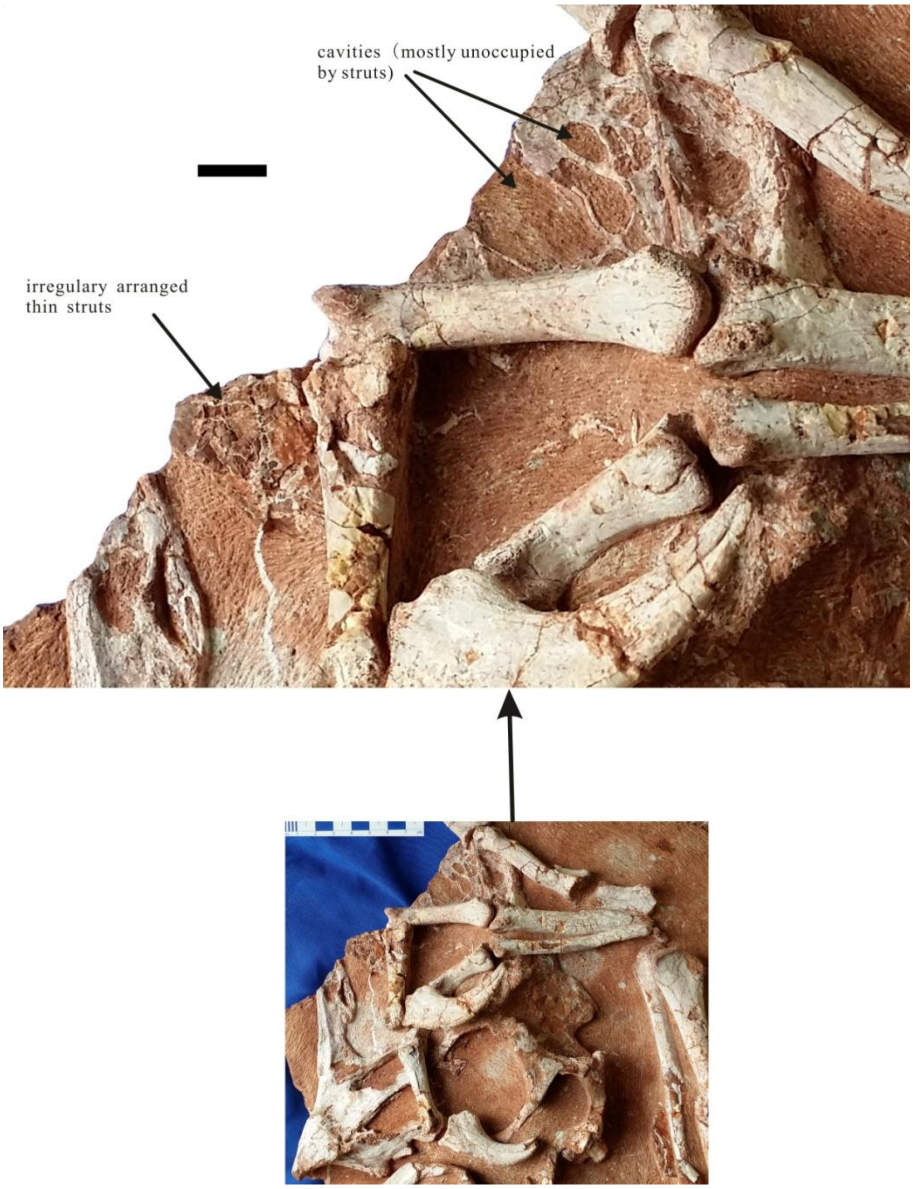

Supplementary Fig. s1. Close up of the skull showing the pneumatic crest.

3. Bone microstructures

Rib (Fig. s2) – A thin section of the rib reveals that cortical and medullary structures underwent a considerable recrystallization and were further eroded by intrusion of the surrounding reddish sediment (Fig. 2B). The cortex consists mostly of the Haversian bone; a thin layer of primary osteons is present only under periosteal surface. The diameter of primary neurovascular canals varies between 11 x 14 µm and 14 x 21 µm. The secondary osteons show variable diameter of central canals (from 20 x 25 µm to 53 x 46 µm; Fig. 2C), some of which had considerably enlarged (diameter up to 112 x 140 µm) due to osteoclastic erosion Fig. s2D). All observed neurovascular canals are oriented longitudinally.

The preserved rib histology documents that secondary osteons replaced almost the entire primary bone. Dominance of the secondary bone tissue demonstrates an advanced stage of structural remodeling of the rib of *Corythoraptor jacobsi*, perhaps due to exposure to increasing biomechanical stresses. Absence of external circumferential layers suggests that the rib tissue was extensively remodeled before the animal reached somatic maturity.

Fibula (Fig. s3) – The fibular thin section exhibits a dense cortex surrounding the medullary cavity. The outer half of the cortical bone is organized in alternating growth lines and zones; five dark growth lines are preserved and these probably represent periods of inhibited development (Supplementary Fig. 3C). A close-up of the growth line includes a dark line (= line of arrested development, LAG) situated between two layers of slightly birefringent avascular tissue (= annulus, an) (Supplementary Fig. s3B). The complete growth line is 18 to 21 µm wide.

The spacing between the growth lines (AB: 87 µm, CD: 97 µm, DE: 74µm) is fairly consistent except the BC zone that is twice (182 µm) as thick as the other zones. The BC zone probably records bone deposition of two successive seasons, whereas a growth line separating them is not visible due to replacement of the primary bone with relatively large secondary osteons (diameters: 53 x 96 µm to 124 x 184 µm). The secondary bone obliterated a great portion of the growth lines A and B. No growth marks are visible between the growth line A and the internal margin of the cortex, although some growth marks likely existed in the region before the bone was remodeled. The bone tissue external to the growth line E is wider that the DE zone itself, but no growth mark is found in this periosteal part of the fibular section. On the other hand, polarized light reveals a dense population of the Sharpey ́s fibers (179 to 220 µm long) obliquely arranged about 100 µm under the periosteal surface (Fig. s3D). The fibers are distributed among well-developed primary osteons. The primary osteonal canals are found right under the periosteal surface, none of these open onto the external surface.

A distinct thin endosteal bone (= internal circumferential layer) occurs around the medullary cavity (Fig. s3E). The endosteal bone is 30 to 43 µm wide. Laminar character of the bone is visible in the polarized light (Supplementary Fig. 3F). It rims the undulated innermost surface of the cortical bone, documenting different rates of erosional activity that probably resorbed an early growth line. Parallel erosional activity occurs also inside of the secondary neurovascular canals where osteoclastic

resorption enlarged diameter of the canals.

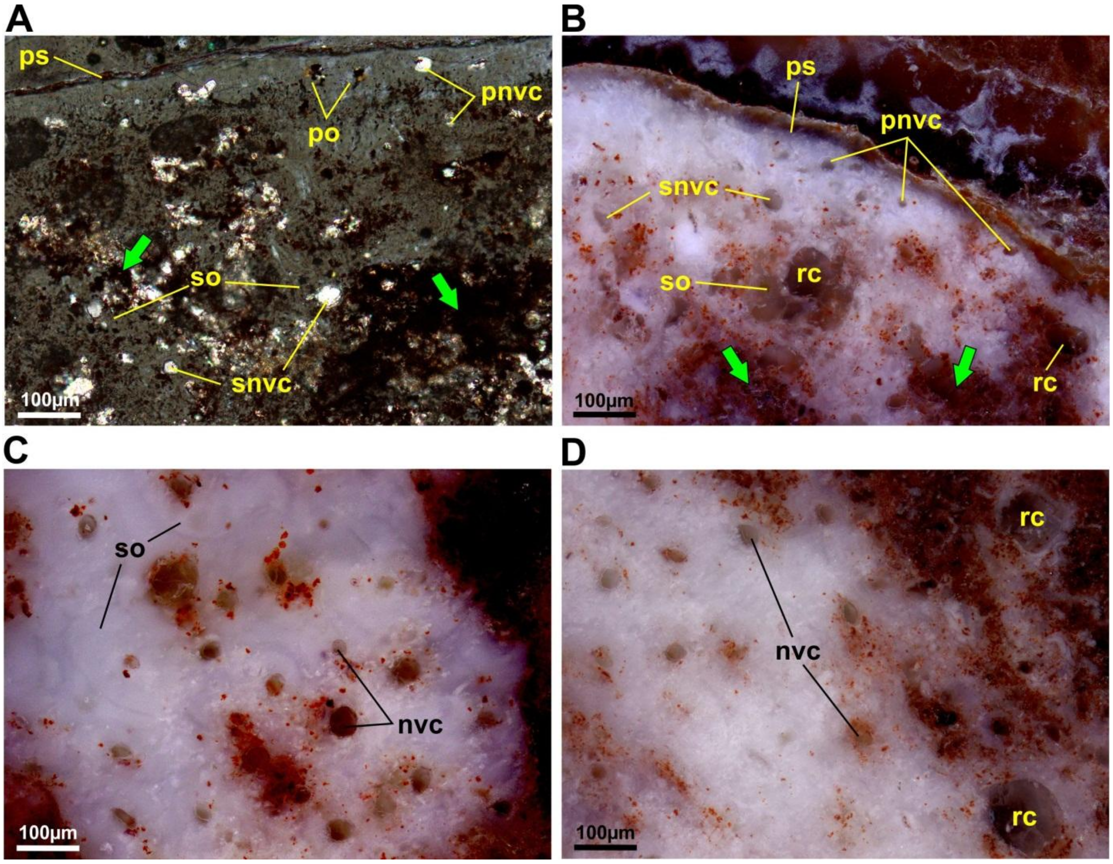

Fig. s2**. Rib microstructure of the holotype of *Corythoraptor jacobsi* gen. et sp. nov.** (JPM-2015-001)**.**

A, Close up of the periosteal cortex (transversal section) viewed with transmitted light. Note extensive corrosion of histostructure and secondary mineral intrusion (green arrows) located throughout the section. **B,** Close up of the periosteal cortex (polished surface sample) viewed with reflected light. Remaining primary osteons preserved at the periosteal surface. Note advanced periosteal succession of secondary osteons, some of which display osteoclastic cavitation (oe). **C,** Intracortical remodelation (transversal section) viewed with reflected light. **D,** Extensive osteoclastic erosion of the neurovascular canals of the Haversian bone tissue. Note original size of secondary neurovascular canals. Abbreviations: nvc, neurovascular canal; pnvc, primary neurovascular canal; po, primary osteon; ps, periosteal surface; rc, resorption cavity; snvc, secondary neurovascular canal; so, secondary osteon.

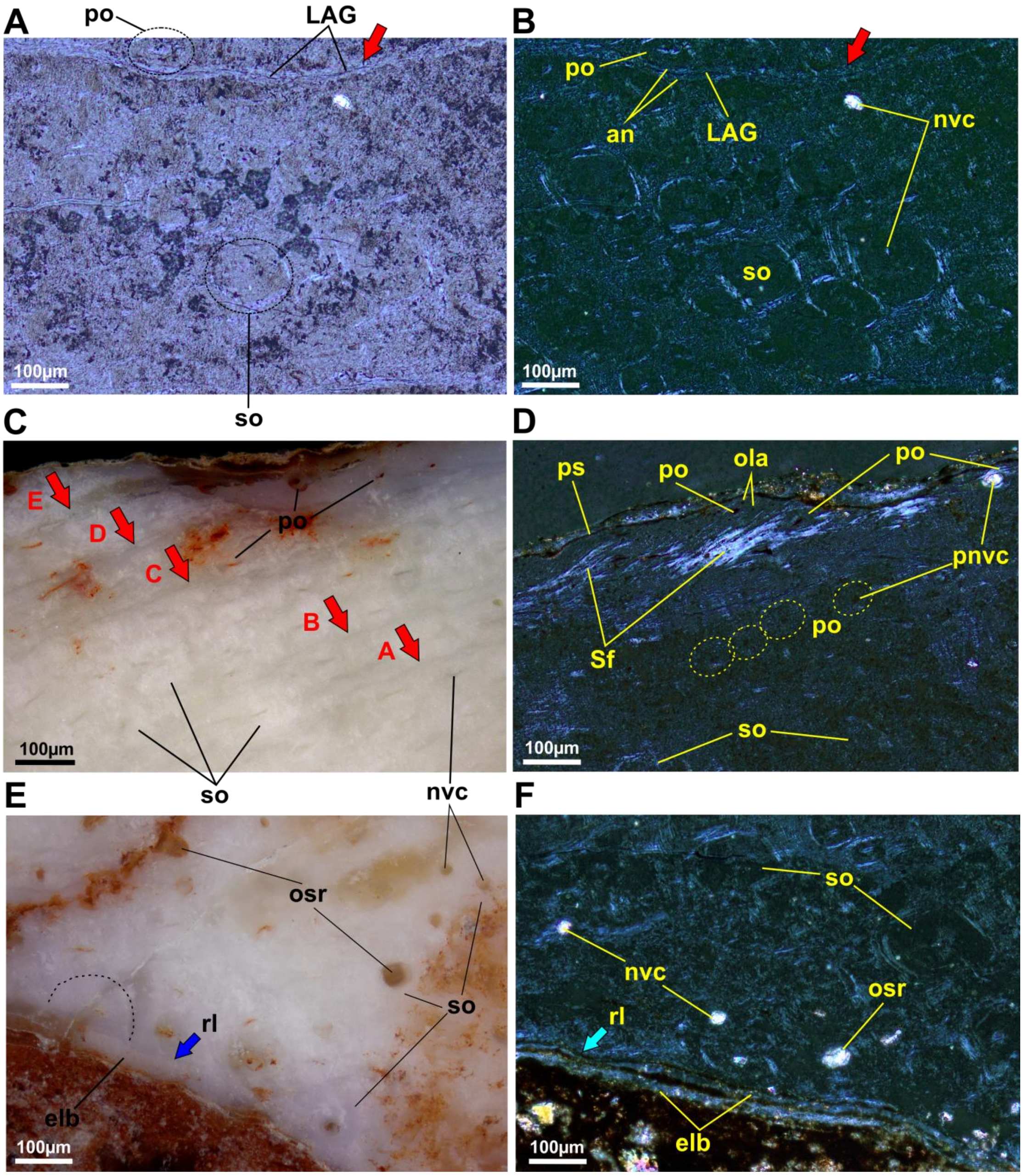

Fig. s3**. Fibula microstructure of the holotype of *Corythoraptor jacobsi* gen. et sp. nov.** (JPM-2015-001)**.**

A, Close up of the intracortical tissue (transversal section) viewed with transmitted light. Note corroded histostructure which osteonal composition is difficult to read; some growth lines are visible (red arrow). **B,** Same region as A in circularly polarized light to highlight more birefringent osteonal lamellae and the tissue (annulus; red arrow) adjacent to the line of arrested development (LAG). **C,** Diaphyseal transversal section in reflected light showing five growth lines (red arrows A to E) present in the middle-to-outer cortical bone. **D,** The transversal

view in circular polarized light elucidates strongly ordered Sharpey´s fibers distributed inside a weakly birefringent primary bone near the periosteal surface. Note the presence of well-developed primary osteons just internal to the periosteal surface; this indicates that this bone was still growing in time when the animal perished. **E,** The inner cortical bone made of secondary osteons (transversal section) in reflected light showing deposition of endosteal lamellar bone. Note partly eroded osteons (black dash line) and the resorption line (blue arrow). Some of secondary neurovascular canals with increased diameter that indicates initial osteoclastic activity. **F,** The transversal view of the inner cortex in circularly polarized light to highlight the presence of more birefringent endosteal lamellae. Note the resorption line (blue arrow). Abbreviations: an, annulus; elb, endosteal laminar bone; LAG, line of arrested growth; nvc, neurovascular canal; ola, osteocyte lacunae; osr, osteonal resorption; pnvc, primary neurovascular canal; po, primary osteon; ps, periosteal surface; rl, resorption line; Sf, Sharpey´s fibers; so, secondary osteon.

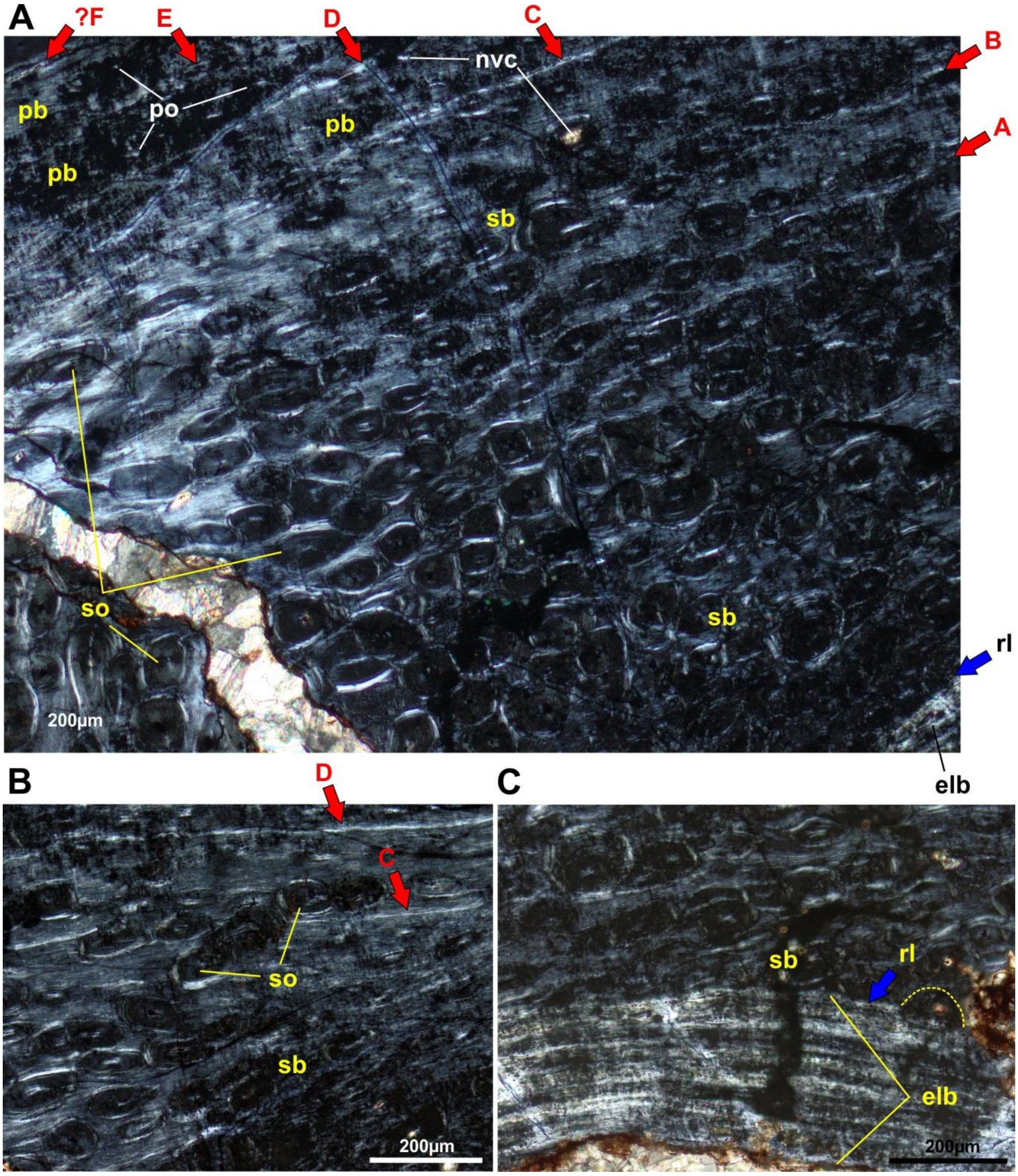

Fig. s4. **Radius microstructure of the holotype of *Corythoraptor jacobsi* gen. et sp. nov.** (JPM-2015-001) **viewed with circular polarized light microscopy.**

A, Transversal cross-section of the diaphysis showing five growth lines (A to E), and the putative sixth growth line (F) near the periosteal surface. Note that the innermost preserved growth line (A) is located in the mid-cortex. The cortex internal to the growth line C consists of the Haversian bone. The inner cortex is well separated from strongly birefringent the endosteal laminar bone by the resorption margin (blue arrow). The marrow cavity lies in the direction to the bottom right. **B,** Close-up of the mid-cortex showing gradual obliteration of the growth line C by secondary osteons. **C,**

Haversian bone of the inner cortex sharply separated (blue arrow) from strongly birefringent laminae of the thick endosteal bone. Note eroded margin of the secondary osteon (yellow dash line). Abbreviations: elb, endosteal laminar bone; nvc, neurovascular canal; pb, primary bone; po, primary osteon; rl, resorption line; sb, secondary bone; so, secondary osteon.

4. Character list and datamatrix for phylogenetic analysis

4.1 Character list (from Lü et al.[s1] and Funston and Currie[s2], based on that of Lamanna et al.[s3]). Characters 238 to 257 are chosen from Funston and Currie[s2] (characters 231 to 250 in Funston and Currie[s2])

1. Ratio of the preorbital skull length to the basal skull length: 0.6 or more (0); less than 0.6 (1) (Lü et al., 2013b).

2. Pneumatized crest-like prominence on the skull roof: absent (0); present (1).

3. Ratio of the width (across premaxilla–maxilla suture) of the snout to its length: less than 0.3 (0); 0.3–0.4 (1); more than 0.4 (2) (Lü et al., 2013b). (ORDERED)

4. Ratio of the length of the tomial margin of the premaxilla to the premaxilla height (ventral to the external naris): 0.7 or less (0); 1.0–1.4 (1); more than 1.7 (2). (ORDERED)

5. Inclination of the anteroventral margin of the premaxilla relative to the horizontally positioned ventral margin of the jugal: vertical (0); posterodorsal (1); anterodorsal (2).

6. Ventral projection of the premaxilla below the ventral margin of the maxilla: absent (0); present (1).

7. Ventral projection of the premaxilla below the ventral margin of the maxilla:

small (0); significant (1)

8. Share of the premaxilla (ventral) in the basal skull length: 0.10 or less (0); 0.12 or more (1).

9. Pneumatization of the premaxilla: absent (0); present (1).

10. Ratio of the length of the maxilla (in lateral view) to the basal skull length: 0.4–0.7 (0); less than 0.4 (1) (Lü et al., 2013b).

11. Subantorbital portion of the maxilla: not inset medially (0); inset medially (1).

12. Palatal shelf of the maxilla with two longitudinal ridges and a tooth-like ventral process: absent (0); present (1).

13. Ventral margins of maxilla and jugal: margins form a straight line (0); the ventral margin of the maxilla slopes anteroventrally, its longitudinal axis at an angle of ca. 120 °to the longitudinal axis of the jugal (1).

14. Rim around antorbital fossa: well pronounced (0); poorly delimited (1).

15. Antorbital fossa: bordered anteriorly by the maxilla (0); bordered anteriorly by the premaxilla (1).

16. Accessory maxillary fenestrae: absent (0); at least one accessory fenestra present (1).

17. Nasal along midline: longer than frontal (0); shorter than or as long as the frontal (1).

18. Nasals: separate (0); fused (1).

19. Subnarial process of the nasal: long (0); short (1).

20. Shape of the narial opening: longitudinally oval (0); teardrop-shaped, slightly

longer than wide (1); much longer than wide (2). (ORDERED)

21. Nasal recesses: absent (0); present (1).

22. External naris position relative to the antorbital fossa: naris and fossa widely separated (0); posterior margin of the naris reaching the fossa (1); overlapping anterodorsally most of the fossa (2). (ORDERED)

23. Ventral margin of the external naris: at the level of the maxilla (0); dorsal to the maxilla (1).

24. Prefrontal: present (0); absent or fused with the lacrimal (1).

25. Lacrimal shaft: not projecting outward beyond the orbital plane and lateral surface of the snout (0); the middle part of the shaft projecting laterally to form a flattened transverse bar in front of the eye (1).

26. Lacrimal recesses: absent (0); present (1).

27. Ratio of the length of the orbit to the length of the antorbital fossa: 0.7–0.9 (0); 1.2 or more (1).

28. Ratio of the length of the parietal to the length of the frontal: 0.6 or less (0); 1.0 or more (1).

29. Pneumatization of skull roof bones: absent (0); present (1).

30. Sagittal crest along the interparietal contact: absent (0); present (1).

31. Supratemporal fossa: invading the frontal (0); not invading the frontal (1).

32.Infratemporal fenestra: dorsoventrally elongate, narrow anteroposteriorly (0); subquadrate, its anteroposterior length comparable to the orbital length (1).

33. Pneumatization of the squamosal: absent (0); present (1).

34. Cotyle-like incision on the ventrolateral margin of the squamosal (for reception of the dorsal end of the ascending process of the quadratojugal): absent (0); present (1).

35. Ventral ramus of the jugal: deep dorsoventrally and flattened mediolaterally (0); shallow dorsoventrally or rod-shaped (1).

36. Jugal process of the postorbital: not extending ventrally below two-thirds of the orbit height (0); long, extending ventrally close to the base of the postorbital process of the jugal (1).

37. Postorbital process of the jugal: posterodorsally inclined (0); perpendicular to the ventral ramus of the jugal (1).

38. Postorbital process of the jugal: present (0); absent (1).

39. Jugal–postorbital contact: present (0); absent (1).

40. Quadratojugal process of the jugal in lateral view: forked (0); not forked (1); fused with the quadratojugal (2).

41. Quadratojugal–squamosal contact: absent (0); present (1).

42. Ascending (squamosal) process of the quadratojugal: bordering ca. the ventral half, or less, of the infratemporal fenestra (0); bordering the ventral two-thirds or more of the infratemporal fenestra (1).

43. Ascending (squamosal) process of the quadratojugal: present (0); absent (1).

44. Angle between the ascending and jugal processes of the quadratojugal: ca. 90 ° (0); less than 90 °(1).

45. Quadrate process of the quadratojugal: well developed, extending posteriorly

or posteroventrally beyond the posterior margin of the ascending process (0); not extending beyond the posterior margin of the ascending process (1).

46. Dorsal part of the quadrate: erect (0); bent backward (1).

47. Otic process of the quadrate: articulating only with the squamosal (0); articulating with the squamosal and the lateral wall of the braincase (1).

48. Pneumatization of the quadrate: absent (0); present (1).

49. Lateral accessory process on the distal end of the quadrate for articulation with the quadratojugal: absent (0); present (1).

50. Lateral cotyle for the quadratojugal on the quadrate: absent (0); present (1).

51. Mandibular condyles of quadrate: posterior to the occipital condyle (0); in the same vertical plane as the occipital condyle (1); anterior to the occipital condyle (2). (ORDERED)

52. Nuchal transverse crest: pronounced (0); not pronounced (1).

53. Occiput position in relation to the ventral margin of the jugal–quadratojugal bar: about perpendicular (0); inclined anterodorsally (1).

54. Paroccipital process: directed laterally (0); directed ventrally (1).

55. Foramen magnum: smaller than or equal in size to the occipital condyle (0); larger than the occipital condyle (1).

56. Basal tubera: modestly pronounced (0); well developed, widely separated (1).

57. Pneumatization of the basisphenoid: weak or absent (0); extensive (1).

58. Basipterygoid processes: well developed (0); strongly reduced (1).

59. Basipterygoid processes:present (0); absent (1).

60. Parasphenoid rostrum: horizontal or anterodorsally directed (0); sloping anteroventrally (1).

61. Depression in the periotic region: absent (0); present (1).

62. Pneumatization of the periotic region: absent or weak (0); extensive (1).

63. Quadrate ramus of the pterygoid: distant from the braincase wall (0); overlapping the braincase (1).

64. Pterygoid basal process for contact with the basisphenoid: absent (0); present (1).

65. Ectopterygoid position: lateral to the pterygoid (0); anterior to the pterygoid (1).

66. Ectopterygoid contacts with the maxilla and lacrimal: absent (0); present (1).

67. Ectopterygoid: short anteroposteriorly with a hook-like jugal process (0); elongate, shaped like a Viking ship, without a hook-like process (1).

68. Massive pterygoid–ectopterygoid longitudinal bar: absent (0); present (1).

69. Palate extending below the cheek margin: absent (0); present (1).

70. Palatine: tetraradiate or trapezoidal (0); triradiate, without a jugal process (1); developed in horizontal, longitudinal, and transverse planes perpendicular to each other (2).

71. Pterygoid wing of the palatine: dorsal to the pterygoid (0); ventral to the pterygoid (1).

72. Maxillary process of the palatine: shorter than the vomeral process (0); longer than the vomeral process (1).

73. Vomer: distant from the parasphenoid rostrum (0); approaching or in contact with the parasphenoid rostrum (1).

74. Suborbital (ectopterygoid–palatine) fenestra: well developed (0); closed or reduced (1).

75. Jaw joint: distant from the midline of the skull (0); close to the skull midline (1).

76. Movable intramandibular joint: present (0); suppressed (1).

77. Mandibular symphysis: loose (0); tightly sutured (1); fused (2). (ORDERED)

78. Extended symphyseal shelf at the mandibular symphysis: absent (0); present (1).

79. Downturned symphyseal portion of the dentary: absent (0); present (1).

80. U-shaped mandibular symphysis: absent (0); present (1).

81. Ratio of the length of the retroarticular process to the total mandibular length: less than 0.05 or the process absent (0); ca. 0.10 (1).

82. Dentary: elongate (0); proportionately short and deep, with maximum depth of dentary between 25% and 50% of dentary length (with length measured from the tip of the jaw to the end of the posterodorsal process) (1); extremely short and deep, with maximum depth 50% or more of dentary length (2) (ORDERED) (Longrich et al., 2013).

83. Ratio of the height of the external mandibular fenestra to the length of the fenestra: 0.2–0.5 (0); 0.7–1.0 (1).

84. External mandibular fenestra: present (0); absent (1).

85. Ratio of the length of the external mandibular fenestra to total mandibular length: absent or not more than 0.10 (0); between 0.15 and 0.20 (1), greater than 0.25 (2). (ORDERED)

86. Process of the surangular dividing the external mandibular fenestra: absent (0); short and broad (1); elongate and spike-like (2) (ORDERED) (Longrich et al., 2010).

87. Coossification of the articular with the surangular: absent (0); present (1).

88. Mandibular rami in dorsal view: straight (0); laterally bowed at midlength (1).

89. Anterodorsal margin of dentary in lateral view: straight (0); concave (1); broadly concave (2) (ORDERED) (Longrich et al., 2013).

90. Posterior margin of the dentary: incised, producing two posterior processes (0); oblique (1).

91. Posterodorsal process of the dentary long and shallow: present (0); absent (1).

92. Posteroventral process of the dentary shallow and long, extending posteriorly at least to the posterior border of the external mandibular fenestra: absent (0); present (1).

93. Coronoid process: posteriorly positioned and vertically projected (0); anteriorly positioned, near the midpoint of the jaw, with a medially hooked apex (1) (Longrich et al., 2013).

94. Surangular foramen: present (0); absent (1).

95. Mandibular articular facet for the quadrate: comprising the surangular and the articular (0); formed exclusively of the articular (1).

96. Mandibular articular facet for the quadrate: with one or two cotyles (0); convex in lateral view, transversely wide (1).

97. Position of the quadrate articular surface relative to the level of the adjoining dorsal margin of the mandibular ramus: ventral (0); moderately elevated, quadrate articulation grades smoothly into remainder of mandible (1); highly elevated, anterior and posterior margins of quadrate articulation at nearly right angles to remainder of mandible (2) (ORDERED) (Lamanna et al. 2014).

98. Anterior part of the prearticular: deep, approaching the dorsal margin of the mandible (0); shallow, strap-like, not approaching the dorsal mandibular margin (1).

99. Splenial: subtriangular, approaching the dorsal mandibular margin (0); strap-like, shallow, not approaching the margin (1).

100. Mandibular adductor fossa: anteriorly delimited, occupying the posterior part of the mandible (0); large, anteriorly and dorsally extended, not delimited anteriorly (1).

101. Coronoid bone: well developed (0); strongly reduced (1).

102. Coronoid bone: present (0); absent (1).

103. Premaxillary teeth: present (0); absent (1).

104. Maxillary tooth row: extends at least to the level of the preorbital bar (0); does not reach the level of the preorbital bar (1); maxillary teeth absent (2). (ORDERED)

105. Dentary teeth: present (0); absent from tip of jaw but present posteriorly (1);

absent (2) (ORDERED) (Longrich et al., 2013).

106. Number of cervicals (excluding cervicodorsal): not more than 10 (0); more than 10 (1).

107. Anterior articular facets of the centra in the anterior postaxial cervicals: not inclined or only slightly inclined (0); strongly inclined posteroventrally, almost continuous with the ventral surfaces of the centra (1).

108. Centra of the anterior cervicals: not extending posteriorly beyond their respective neural arches (0); extending posteriorly beyond their respective neural arches (1).

109. Epipophyses on the postaxial cervicals: in the form of a low crest or rugosity (0); prong-shaped (1).

110. Cervical ribs in adults: loosely attached to vertebrae (0); firmly attached (1); fused (2). (ORDERED)

111. Shafts of cervical ribs: longer than their respective centra (0); not longer than their respective centra (1).

112. Lateral pneumatic fossae (‘pleurocoels’) on the dorsal centra: absent (0); present (1).

113. Ossified uncinate processes on the dorsal ribs: absent (0); present (1).

114. Number of vertebrae included in the synsacrum in adults: not more than 5 (0); 6 (1); 7–8 (2). (ORDERED)

115. Sacral spines in adults: unfused (0); fused (1).

116. Lateral pneumatic fossae on the sacral centra: absent (0); present (1).

117. Transition point on the caudals: absent (0); present (1).

118. Number of caudals with transverse processes: 15 or more (0); fewer than 15 (1).

119. Lateral pneumatic fossae on the caudal centra: absent (0); present at least in the anterior part of the tail (1).

120. Neural spines confined to: at least 23 anterior caudals (0); at most 16 anterior caudals (1).

121. Number of caudals: more than 35 (0); 30 or fewer (1).

122. Posterior caudal prezygapophyses: overlapping less than half of the centrum of the preceding vertebra (0); overlapping at least half of the centrum of the preceding vertebra (1).

123 Hypapophyses in the cervicodorsal vertebral region: absent (0); small (1); prominent (2). (ORDERED)

124. Posterior hemal arches: deeper than long (0); longer than deep (1).

125. Ratio of the length of the scapula to the length of the humerus: 0.7 or less (0); 0.8-1.1 (1), 1.2 or more (2). (ORDERED)

126. Acromion: projecting dorsally (0); projecting anteriorly (1); everted laterally (2).

127. Posteroventral process of the coracoid: absent or short, not extending beyond the glenoid diameter (0); long, posteroventrally extending beyond the glenoid (1).

128. Orientation of the glenoid on the pectoral girdle: posteroventral (0); lateral (1).

129. Deltopectoral crest: low, its width equal to, or smaller than, the shaft diameter (0); expanded, wider than the shaft diameter (1).

130. Extent of the deltopectoral crest (measured from the humeral head to the apex of the crest): about the proximal third of the humerus length or less (0); ca. 40%–50% of the humerus length (1).

131. Shaft of the ulna: straight (0); bowed, convex posteriorly (1).

132. Ratio of the length of the radius to the length of the humerus: 0.80 or less (0); 0.85 or more (1).

133. Combined lengths of manual phalanges III-1 and III-2: greater than the length of phalanx III-3 (0); less than or equal to the length of phalanx III-3 (1).

134. Ratio of the length of metacarpal I to the length of metacarpal II: 0.5 or more (0); less than 0.5 (1).

135. Proximal margin of metacarpal I in dorsal view: straight, horizontal (0); angled due to a medial extent of the carpal trochlea (1).

136. Metacarpal II relative to metacarpal III: shorter (0); subequal (1); longer (2). (ORDERED)

137. Ratio of the length of metacarpal II to the length of the humerus: 0.4 or less (0); more than 0.4 (1).

138. Ratio of the length of the manus to the length of the humerus plus the radius: less than 0.50 (0), between 0.50 and 0.65 (1), greater than 0.65 (2). (ORDERED)

139. Ratio of the length of the manus to the length of the femur: 0.3–0.6 (0); more than 0.7 (1).

140. Ratio of the length of the humerus to the length of the femur: 0.50–0.69 (0); 0.70 or more (1) (Lü et al., 2013b).

141. Dorsal margins of opposite iliac blades: well separated from each other (0); close to or contacting each other along their medial sections (1).

142. Dorsal margin of the ilium along the central portion of the blade: straight (0); arched (1); concave (2) [modified based on *Nomingia*]

143. Preacetabular process of the ilium relative to the postacetabular process (lengths measured from the center of the acetabulum): shorter or equal (0); longer (1).

144. Preacetabular process: not expanded or weakly expanded ventrally below the level of the dorsal acetabular margin (0); expanded ventrally well below the level of the dorsal acetabular margin (1).

145. Morphology of the ventral margin of the preacetabular process: cuppedicus fossa absent, margin transversely narrow (0); cuppedicus fossa or a wide shelf present (1); margin flat, wide at least close to the pubic peduncle (2).

146. Anteroventral extension of the preacetabular process: absent (0); present (1).

147. Anteroventral extension of the preacetabular process: with rounded tip (0); hook-like (1).

148. Posterior end of the postacetabular process: truncated or broadly rounded (0); narrowed or acuminate (1).

149. Anteroposterior length of the pubic peduncle: about the same as that of the ischial peduncle (0); distinctly greater than that of the ischial peduncle (1).

150. Dorsoventral extension of the pubic peduncle: level with the ischial peduncle (0); deeper than the ischial peduncle (1).

151. Ratio of the length of the ilium to the length of the femur: 0.50–0.79 (0); 0.80 or more (1) (Lü et al., 2013b).

152. Pelvis: propubic (0); mesopubic (1); opisthopubic (2). (ORDERED)

153. Pubic shaft: straight (0); concave anteriorly (1).

154. Pubic foot: anterior process absent or shorter than posterior process (0); two processes equally long (1); anterior process longer than posterior process (2). (ORDERED)

155. Posterior margin of the ischial shaft: straight or almost straight (0); distinctly concave (1).

156. Greater trochanter of the femur: weakly separated, or not separated, from the femoral head (0); distinctly separated from the femoral head (1).

157. Anterior and greater trochanters: separated (0); contacting (1).

158. Dorsal extremity of the anterior trochanter: well below the greater trochanter (0); about level with the greater trochanter (1).

159. Fourth trochanter: well developed (0); weakly developed or absent (1).

160. Adductor fossa and the associated anteromedial crest on the distal femur: weak or absent (0); well developed (1).

161. Distal projection of the fibular condyle of the femur beyond the tibial condyle: absent (0); present (1).

162. Ascending process of the astragalus: as tall as wide across the base (0); taller

than wide (1).

163. Distal tarsals: not fused with the metatarsus (0); fused with the metatarsus (1).

164. Proximal coossification of metatarsals II–IV: absent (0); present (1).

165. Arctometatarsus: absent (0); present, but only proximalmost extreme of metatarsal III obscured from anterior view in articulated metatarsus (1); present, proximal ~half of metatarsal III obscured from anterior view in articulated metatarsus (2) (Lamanna et al., 2014). (ORDERED)

166. Length of metatarsal I constituting: more than 50% of metatarsal II length (0); less than 50% of metatarsal II length (1); metatarsal I absent (2). (ORDERED)

167. Ratio of the maximum length of the metatarsus to the length of the femur: less than 0.3 (0), between 0.4 and 0.6 (1), 0.7-0.8 (2). (ORDERED)

168. Crenulated tomial margin of the premaxilla: absent (0); present (1).

169. Frontals: flat or weakly arched, not strongly projecting above orbit in lateral view (0); strongly arched, projecting well above orbit in lateral view to contribute to nasal–frontal crest (1).

170. Exoccipital: short, weakly projecting (0); strongly projects ventrally beyond squamosal in lateral view, approaching ventral end of quadrate (1).

171. Dentary posterodorsal ramus: straight or weakly curved (0); strongly bowed dorsally (1).

172. Dentary symphyseal ventral process: absent (0); prominent process present on posteroventral surface of symphysis (1).

173. Dentary anteroventral margin in lateral view: straight or weakly downturned (0); strongly downturned (1).

174. Lateral surface of dentary: smooth (0); bearing a deep fossa, sometimes with associated pneumatopore (1).

175. Angular: contributes extensively to the border of the external mandibular fenestra (0); largely excluded by surangular (1).

176. Surangular with an anteroposteriorly elongate flange on the ventral edge: absent (0); present (1).

177. External mandibular fenestra: elongate (0); height subequal to length (1).

178. Dentary contribution to external mandibular fenestra relative to length of dentary: no more than 50% (0); exceeds 50% (1).

179. Metacarpal I expanded ventrally to cover ventral surface of metacarpal II: absent (0); present (1).

180. Unguals of manual digits II and III: strongly curved (0); weakly curved (1).

181. Manual phalanx I-1: slender (0); more robust than II-1 (1); more than 200% diameter of II-1 (2) (ORDERED).

182. Manual phalanx III-3: longer than phalanx III-2 (0); shorter than or equal in length to III-2 (1).

183. Manual phalanx II-2: longer than II-1 (0); subequal to or slightly shorter than II-1 (1); distinctly shorter than II-1 (2) (ORDERED) (Longrich et al., 2013).

184. Manual digit II: elongate, with combined lengths of manual phalanges II-1 and II-2 longer than metacarpal II (0); combined lengths of manual phalanges II-1

and II-2 subequal to metacarpal II (1) (Lamanna et al., 2014).

185. Ischium strongly bent posteriorly at midshaft, distal end forms an angle of at least 60 °with proximal end: absent (0); present (1).

186. Metatarsus: elongate (0); short, length does not exceed 300% of proximal width (1).

187. Ilium: tall (0); low and anteroposteriorly elongate, height less than 25% of length (1).

188. Anterior blade of ilium shallower than posterior blade: absent (0); present (1).

189. External naris: placed anteriorly (0); extends posteriorly, with posterior end lying above antorbital fenestra (1).

190. Premaxillae, nasal processes anteroposteriorly expanded and mediolaterally compressed to form a bladelike internarial bar: absent (0); present (1).

191. Dentary, anterodorsal tip of beak: projecting upwards (0); projecting anterodorsally, tip of beak projecting at an angle of 45 ° or less relative to the ventral margin of the symphysis (1).

192. Dentary symphysis with interior surface bearing vascular grooves and associated foramina: absent (0); present (1).

193. Dentary symphysis bearing an hourglass-shaped ventral depression: absent (0); present (1).

194. Meckelian groove terminates: on the inside of the dentary (0); on the ventral surface of the symphysis (1).

195. Lingual triturating shelf: absent (0); present (1).

196. Symphyseal ridges inside the tip of the beak: absent (0); present but weakly developed (1); present and well developed (2) (ORDERED).

197. Lingual ridges inside the lateral occlusal surface of beak: absent (0); present (1).

198. Posteroventral process of dentary: straight (0); bowed ventrally (1).

199. Dentaries pneumatized: absent (0); present (1).

200. Dentary: participates in dorsal border of the external mandibular fenestra (0); excluded by anterior extension of the surangular (1).

201. Dentary: participates in ventral border of external mandibular fenestra (0); excluded by anterior extension of the angular (1).

202. Surangular and angular divided by posterior extension of the external mandibular fenestra: absent (0); present (1).

203. Posterior end of the surangular: deep (0); shallow, subequal to or shallower than angular (1).

204. Surangular: deep anteriorly (0); strap-like (1).

205. Retroarticular process extends: posteriorly (0); posteroventrally (1).

206. Metacarpal I: proportionately broad (0); long and slender, diameter 20% of length (1).

207. Manual phalanx I-1: longer than II-2 (0); subequal to II-2 (1); shorter than II-2 (2) (ORDERED).

208. Ischiadic peduncle of pubis with prominent medial fossa: absent (0); present (?).

209. Ischium, obturator process located: distally (0); at midshaft of ischium (1).

210. Anterior margin of obturator process: straight or convex (0); distinctly concave (1).

211. Accessory trochanter of femur: weakly developed (0); prominent, subrectangular flange or finger-like process (1).

212. Metatarsal III: with an ovoid or subtriangular cross section (0); anteroposteriorly flattened, with a concave posterior surface (1).

213. Paroccipital process: elongate and slender, with dorsal and ventral edges nearly parallel (0); short and deep with convex distal end (1).

214. Mandibular articulation surface: as long as ventral end of quadrate (0); twice or more as long as quadrate surface, allowing anteroposterior movement of mandible (1).

215. Scars for interspinous ligaments in dorsal vertebrae terminate: at apex of neural spine (0); ventral to apex of neural spine (1)

216. Sternum, distinct lateral xiphoid process posterior to costal margin: absent (0); present (1)

217. Anterior edge of sternum: grooved for reception of coracoids (0); without grooves (1)

218. Deltopectoral crest: large and distinct, proximal end of humerus quadrangular in anterior view (0); less pronounced, forming an arc rather than being quadrangular (1).

219. Ischium: more than two-thirds of pubis length (0); two-thirds or less of pubis

length (1)

220. Lateral ridge of femur: absent or represented only by faint rugosity (0); distinctly raised from shaft, mound-like (1)

221. Surangular, distinct groove on dorsal surface: present (0); absent (1).

222. Vomer, position: level with other palatal elements (0); ventral to other palatal elements (1)

223. Calcaneum: excludes astragalus from reaching lateral margin of tarsus (0); small process of astragalus protrudes through a circular opening in edge of calcaneum to reach lateral margin of tarsus (1).

224. Depression on lateral surface of dentary immediately anterior to external mandibular fenestra: absent (0); present (1).

225. Groove on ventrolateral edge of angular to receive posteroventral branch of dentary: absent (0); present (1).

226. Posteroventral branch of dentary twisted so that lateral surface of branch faces somewhat ventrally: absent (0); present (1).

227. Premaxilla, large, presumably pneumatic foramen at anteroventral corner of narial fossa: absent (0); present (1).

228. Accessory opening at anterodorsal extreme of snout: absent (0); present (1).

229. Development of symphyseal shelf of mandible: limited, anteroposterior length of mandibular symphysis (as measured on midline) less than 20% total anteroposterior length of mandible (0); intermediate, length of symphysis greater than 20% but less than 25% length of mandible (1); extensive, length of symphysis

greater than 25% mandibular length (2) (ORDERED).

230. Prominent flange or shelf arising from lateral surface of dentary: absent (0); present (1).

231. Base of retroarticular process: considerably wider mediolaterally than tall dorsoventrally (0); approximately as wide as tall (1); considerably taller than wide (2). (ORDERED)

232. Posteriormost caudal vertebrae fused, forming a pygostyle-like structure: absent (0); present (1).

233. Humeral shaft: straight or nearly straight (0); strongly bowed laterally (1).

234. Proximodorsal extensor 'lip' on manual unguals: weak (i.e., continuous or nearly continuous with remainder of dorsal surface of ungual) and/or absent (0); prominent ('set off' from remainder of dorsal surface by distinct change in slope immediately distal to 'lip') (1).

235. Pubic process of ischium, 'hooked' anterodistal extension: absent (0); present (1).

236. Distal margin of obturator process: straight (0); distinctly concave, apex of obturator process angled distally (1).

237. Proximolateral edge of metatarsal IV attenuated into pointed process: absent (0); present (1).

238. Frontal anteriorly divided by slot for nasal and possibly lacrimal: absent (0); present (1).

239. Infradiapophyseal infraprezygapophyseal and infrapostzygapophyseal fossae

on cervical and dorsocervical vertebrae: one or more absent (0); all three present (1).

240. Ratio of minimum shaft diameter of manual phalanx II-1 to minimum shaft diameter of metacarpal II: greater than 1 (0); smaller than 1 (1).

241. Ratio of minimum shaft diameter to length of manual phalanx II-2: greater than 0.10 (0); smaller than 0.10 (1).

242. Ratio of the length of the metatarsus to the length of the tibia: <0.5 (0); >0.5 (1).

243. Tibia ratio of the transverse width of the distal condyles to the length: 0.20 or greater (0); <0.20 (1).

244. Ratio of minimum transverse width to length of tarsometatarsus: >0.20 (0); <0.20 (1)

245. Fusion of distal tarsals III and IV at maturity: absent (0); present (1).

246. 'Hook-like' posterodorsal process of distal tarsal IV: absent (0); present (1).

247. Posterior protuberance on proximal end of tarsometatarsus caused by coossification of distal tarsals III and IV, plus MT II, III and IV: absent (0); present (1).

248. Anterior margin of metatarsal V in lateral view: straight or slightly curved (0); tightly curved (1).

249. Concavity on posterior surface of tarsometatarsus in cross section: absent or shallow (0); prominent and deep (1).

250. Sharp cruciate ridges on posterior surface of metatarsal III: only one sharp

longitudinal ridge or no ridges (0); sharp medial longitudinal ridge continuous with lateral postcondylar ridge and sharp lateral longitudinal ridge continuous with medial postcondylar ridge forming a chiasmata. ridges are separated from each other by longitudinal sulcus (1).

251. Posteromedial and posterolateral ridges of mt II and IV respectively: weakly developed do not extend past posterior extent of distal condyle (0); well developed extend posteriorly past distal condyle (1).

252. Distal ends of shafts of metatarsals II and IV: both straight (0); metatarsal II medially deflected metatarsal IV straight (1); metatarsal II straight metatarsal IV laterally deflected (2).

253. Ratio of transverse width to anteroposterior length of distal condyle of metatarsal III: <1 (0); >1 (1).

254. External mandibular fenestra: expanded anteriorly (0); anteriorly constricted by posteroventral ramus of dentary (1).

255. Articular ridge of mandible: low, less than 25% as tall as long (0); high, more than 25% as tall as long (1).

256. Transverse groove between flexor tubercle and proximal articular surface of manual ungual I-2: absent (0); present (1)

257. Cnemial crest of tibia greatly enlarged such that the proximal articular surface of the tibia is longer anteroposteriorly than wide mediolaterally: absent (0); present (1).

4.2 Datamatrix

Herrerasaurus_ischigualastensis
000100-0000000000000000000000000000000000000000000000000000000
000000000000000000100000000000000000000000010?000000000100?0000101??0
00100000000-00001010000000000000100000?000?0000110010100??000??0000101?
?00??0000???00?1000?000000?00000???????000?0000?000?
Velociraptor_mongoliensis
000200-00000010100101001010001000000000001000000000100001000000000
0000000000000000000000011000000000000010010110111101101011111-110101112
02110000110111120200111000100100000000000?0000000011000?0000??001000010
0000000110011?000?00000000000000?????00000000?000?
Archaeopteryx_lithographica
000?10-000000101101001100010001?1000-1110??0?1??0020????000????00000
01?1???1000010-10000011-01000000-10100??0101?00?11011001011100111111111
011010-111020201?0100?1101200?0000?0?0?00000001000?0000??001?000000?010
010?0?111??00?00000?0000?00??????00000000?000?
Incisivosaurus_gauthieri
100210-101000011?0111210011?01?00?00000?100010?0??200111?10?10?01??1
11?0101?2101110020??00011??11?1110011?????????????????????????????????????
?????????????????????????00000000100??????????001000000000000000???????1???
????00???01000???????0????????????????000?
Caudipteryx_zoui
10?210-?01??0?1110?0?21?01?10???011000011100100????????????????????0???
?????0??1111??2???0001??????11??0220?????010????0?1??11???1011?1010200?00
12110110?021100???1000121??0???0??0?00?001000?000?00000000?01000?0010??
?0101????0?00000?0000000?0??????000000?000?
Avimimus_portentosus
????????????0???????????????10110??1?-1121-101??00?101111?10????????????
???0?1??111???01?????11000???-11?21000110?110??0???2?12100111?1?????00000
?1110?10020101001111222100010000?0???????00??00?00????0??0000??0??10110?
?11110????????0?0?000010?????1000000?00??
Microvenator_celer
??
??1111?2?????11001?????????????2???0??1?????????0????1?1011??1????0???1111?1
1?112???11101????????0101???001[0
1]?????00??0000000?0??????0?1??1???0??1?0???1?????0??00???????????????????
???
Oviraptor_philoceratops
1????????????1?11?????21?????????1??10000????0??????????????????????????1???
??????1?121022??10011???1??1???22???0??1?????????????2???101101?212????????
??????????????????????0???001001000000??0??1?????????????????0???????????0??1

1?0?0???0??00???0???????????????000?
Rinchenia_mongoliensis
11102??0111110111???1211111111?1111010011100011111?11111-11?11111111 2111111111112102?01100111111111-11221110011?1010010102?22101000??????01 1011111101????111111??????1101?001011????????00111??????00000001????????1? 10???1110?0?0?0???????????????????????000?
Citipati_osmolskae
11202111111110110111121111111111111100111011?1??11111??101111?11111 21?11111111121022011001111111110122???????1????????????????0?101?1??????? ????????????????????????11011001011?01010?0??110000000000000100?????0101 00?01110?001000100?0?0??????00?0??00?000?
Zamyn_Khondt_oviraptorid
112021111111101111112111111111111110011101111111111111-11111111111 2111111111112102201100111111111-11221110111?10100101021021010110111210 10012101001112111111110001111101?001011?0000010??110?0000000000?0100???0 0?1?100??11???001?0??00??00???????????????000?
Khaan_mckennai
10201101?1111011?1121211111111111?110000111000?111?111111???0??1?????1 ??????1111112102101100111111111???1221110??1????00?0102??210??1101011210? 0001100001?1211111?11000111001100100100101001111110?000?00100000010000000 ?01?1100?1?10101000?0000[0 1]00??????00000000?000?
Conchoraptor_gracilis
101011011111101111121111111111111000011100?1111111?1111-11111111111 21111111111?102?0110011111111211221110111?201001010112210111100111210 1000210000111211111111000111101?????????110???1??11????????????????0??????1 1?00?011?0?0000011?0??000?????00000000?000?
Machairasaurus_leptonychus
?? ?????????????????????????????2???????????????????????????0011??????????????? ???????????????????????????111110?????????????????????00????????????????????? ?????1????????????????????????
Nemegtomaia_barsboldi
1110010111111011111121011111111?1100001010101 1?111111?1??11?1111111 2111111111112102?11100111111?11-11221110[0 2]11?201?????????????????????????10002???00?1????11?????????10111101011?12?1 1??1111000000001000000001000??0??1???1??11?1?1?0001?000??00??????????????00 0?
Heyuannia_huangi
????????????????????????????????????1?????????????????????????????????????? ?????1??21021??1001????1???????21???21-12????1?????221?11000001?20???01?1 0100111?1?????????????????1?1?1011112?2101?1??????????0?00000100?0?0?0101?00 ?1?11?0???0?00001????????000000000000?
Ingenia_yanshini
????????1????????????????11??????1?1100001110?0111111???111???11?1??111?1

???11111112102?01100111111111-1122?110011?2010010101022101100001100001O
012100001112111111110001l?011?10101111212101111100000000000000100?0000?
1?100001?1??0???0?000010?00000000000000?000?
Gigantoraptor_erlianensis
??
11211112002011100111112?????1?2???????????0010?0??10?00000?11?1??1???????
?????1??1111111000?1???0001010000??????????0???000100111010O???0??????1??
???110??112?10??????????010?000??011?
Caenagnathasia_martinsoni
??
??21?1?1?????12????????????????2???
???????????????????0001???0????????????0111111?1?????????????????????????????
??0????????0?????????????????
Elmisaurus_elegans
??
???
????????????1[0
1]1?????????????????????0??????????????????????????1??????????????????????????11?
111???????????????
Leptorhynchos_gaddisi
??
??21?1?[1 2]?????1[1
2]????????????????2???10
1??????0???????????????????0111121?1??????????????????????????????????????1???
??111100111111??1
Chirostenotes_pergracilis
??
??111??????????10?????0????1??11??11110
1???11??1?1100111????????????000101000????????????????12?1111?????????????
????????1000??1111000011001001 10
Caenagnathus_collinsi
??
112101110020112001111111111-1??2???
?????????????????????00010100????????????1101121011111111???????????????1??0
10??201????????????????0???0??000?
Anzu_wyliei
?1??10-1100?1100??????????????????0?0000???????1011??100?10??1??0?011?
????1121011?002011200111111?1-11221110211?1?10010?02?12100010????1210??
?1?11?1?110211111111?????1?000010100000??01?0??0?101121110111111??1111?11
0101101?00101021201110??????1????0?????0000
Hagryphus_giganteus
??
???0010???????????????
????????????????????????????000010??????????????????????2?????????????????????

?????1?????00???????????????1?
Elmisaurus_rarus
??
??001?????????????????
???????????101??????????????000010?0???????????????????11???10???????????????
??????1??1?1??111111011120??11
Nomingia_gobiensis
??
???????????????????????????????????00?1?01?00101020????????????????111011001
1111111111111???????????????????????1?00????????????????????111???0???1???1??
??????1??10?????????????????????
Epichirostenotes_curriei
??????????01?010??????1??????????????????????????????110?10?1??0????????
??????????????????????????????????????0?1?111??1???2??????????????????????????
???021?????????????????????????????1???????????????????????111??0?0???1??????
?????????10??0??????????????????
Alberta_dentary_morph_3
??
?12111?1?????1200??????????????2??
????????????????????0001???0?????????????0111121?10????????????????????????0??
???0?????????????????????????????
Caenagnathus_sternbergi
??
11211111002011200111112?11-2??2??
?????????????????????00010100?????????????111112111111111???????????????1??0
10???0??????????????????????????
Macrophalangia_canadensis
??
??
???????????0011????????????????????0??
????????0????????????????????
Banji_long
11?010-111???0111102121111111011??111001?????0??11?1111?????????111112
1?0111??11?21021??000110??1?11??122?1??
??????????????????????11111001011???????????110??????0?00000????????????????
00?0?000?0???????0???????????????????
Caudipteryx_dongi
??
?????????????????????????????????????01?0?????????????0010?1?10200?011?1101
1000211?????100012????????????00?001000?????????????????01?10?????0??1????
?????????00000???????000000000????
Ganzhousaurus_nankangensis
??
?1[0 1]111?210?[1

2]011001?????111????2?????????????0???0??????????????????????????????????????
??0001????1?001?11???????1????????0?001?10?0???????0???????????111???0?????
?????????000000000????

Jiangxisaurus_ganzhouensis

1??????????1?????????????11???????1?0001????????112??????1-1?????1?01121?
?011111112102111100111111?????122??10??11??1??1?????221?1110?0120????0???
??1???????????????????1??110010111?2?????????111??2?0100000100??????1?1?0?
????0011?00??00???????????????????????

Nankangia_jiangxiensis

??
?11101?2??????100?????????????2???????1???1??1??0?0111001?????????011101101
11111211001111????????1101??0???????1?00??11?????0?1???1???11???????01???
11?0???0??0?00?????????????????????

Ojoraptorsaurus_boerei

??
??
?02??1????????????????????
?????????????????????????????

Shixinggia_oblita

??
??????????????????????????????????????1?2?1??1?????????????????????110000-000
?????1????1??????????????????????????01???????????????????????????????????????
??????????????????????????????

Similicaudipteryx_yixianensis

??
????????????????????????????????????0?11?0?1?0??1?2????????1????????0?001?11111
0?????????0??0?12????????????????????00???????????????????????0??????????????
?????10?????0?????000?0000?????

Wulatelong_gobiensis

11?02101?11?1?1???02121?111?1??1?1110001???00??11?????????????????1111?
???????????????101????1??11?????12????????1???100????2???????????1??????1100?
1111111121???????000?11?????????????0??????011?????????????000????1????1?10?10
1?????????????0??0??0????????00000????

Yulong_mini

102110-11101101101111210101110l0?0100001110010?111110110???0????????
0?????1111111210220110011[0 1]1111?1-2122?100??1????001?10??[1
2]??0000101?11110?110?100??0??1?1?0??11?001110?11001011?11000?100110????
??0?0000010????????1??????1110?01000??00???0??????000?00000????

Huanansaurus_ganzhouensis

112021111111101101?1121111111111?11000110111?11100111?????1???11?11
?????1111111210220110011111?????122?110211????????????????001101?112?????
???????????????????????11011000011?01000????1?1?0????0100000110?????01???
0??1??011??101?01????0?????????0000?1100

Tongtianlong_limosus

10202111111101?0112121111111011111100011000011111?21110?????011?????
1?????111101121021011001101 1??????1221110201??01?01??0?02?1001?0???????0
1??????????1121??????1000?111010001001??????11??111??????01000001???10?10
1?010??1?100010001?0??0000??0??0??00??2?1??0
*Corythoraptor*_jacobsi
112111111111001???21211111?1??01?11000????????????2?1????????????????1?
????111111?2102???0001??????1???1221110211?111??1???2?????001101?11100110
11111011112111111?1001111?11011011001010100011100000001000?0?00?1000??
???110???0110110??01010?0000?100000002?10?0
Apatoraptor_pennatus
???0?1?
??121111100201110011111111-1??211002111?????????2?1111001001120211???101
0???????????101?????????00010100010?10??????01?11?11111111110??????1?101?1
1??011??101?01????111????????????1001

5. Supplementary Fig. 5.

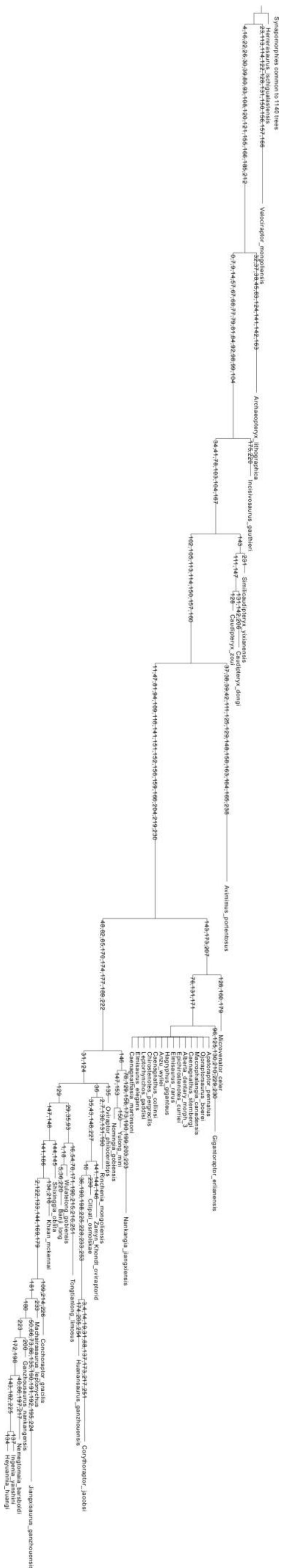

Fig. S5. Strict consensus of 1140 most parsimonious trees with common synapomorphies mapped.

6. References

S1. Lü, J.C., Chen R.J., Brusatte3 S.L., Zhu, Y. X. and Shen C. Z. 2016. A Late Cretaceous diversification of Asian oviraptorid dinosaurs: evidence from a new species preserved in an unusual posture. Scientific Reports, 6:35780, DOI: 10.1038/srep35780

S2. Funston, G. F. and Currie, P.J. 2016. A new caenagnathid (Dinosauria: Oviraptorosauria) from the Horseshoe Canyon Formation of Alberta, Canada, and a reevaluation of the relationships of Caenagnathidae. Journal of Vertebrate Paleontology. DOI: 10.1080/02724634.2016.1160910.

S3. Lamanna, M. C., Sues, H. D., Schachner, E. R. & Lyson, T. R. A new large-bodied oviraptorosaurian theropod dinosaur from the Latest Cretaceous of Western North America. *PLoS ONE* **9,** e92022 (2014).
